# The p53 activator overcomes resistance to ALK inhibitors by regulating p53-target selectivity in ALK-driven neuroblastomas

**DOI:** 10.1038/s41420-018-0059-0

**Published:** 2018-05-10

**Authors:** Makoto Miyazaki, Ryo Otomo, Yuko Matsushima-Hibiya, Hidenobu Suzuki, Ayana Nakajima, Naomi Abe, Arata Tomiyama, Koichi Ichimura, Koichi Matsuda, Toshiki Watanabe, Takahiro Ochiya, Hitoshi Nakagama, Ryuichi Sakai, Masato Enari

**Affiliations:** 1 Division of Refractory and Advanced Cancer, National Cancer Center Research Institute, Chuo-ku, Tokyo 104-0045 Japan; 2 Department of Computational Biology and Medical Sciences, Laboratory of Clinical Genome Sequencing, Graduate School of Frontier Sciences, The University of Tokyo, Minato-ku, Tokyo 108-8639 Japan; 3 Department of Computational Biology and Medical Sciences, Tumour Cell Biology, Graduate School of Frontier Sciences, The University of Tokyo, Minato-ku, Tokyo 108-8639 Japan; 4 Division of Brain Tumour Translational Research, National Cancer Center Research Institute, Chuo-ku, Tokyo 104-0045 Japan; 5 Department of NCC Cancer Science, Medical Research Institute, Tokyo Medical and Dental University (TMDU), 1-5-45 Yushima, Bunkyo-ku, Tokyo 113-8510 Japan; 6 Molecular and Cellular Biology Laboratory, Graduate school of Medical Life Science, Yokohama City University, Tsurumi-ku, Yokohama, Kanagawa 230-0045 Japan; 7 Department of Neurosurgery, National Defense Medical College, 3-2, Namiki, Tokorozawa, Saitama, 359-8513 Japan; 8 Division of Molecular and Cellular Medicine, National Cancer Center Research Institute, Chuo-ku, Tokyo 104-0045 Japan; 9 Division of Cancer Development System, National Cancer Center Research Institute, Chuo-ku, Tokyo 104-0045 Japan; 10 Division of Biochemistry, Kitasato University School of Medicine, 1-15-1 Kitasato, Minami-ku, Sagamihara, Kanagawa 252-0374 Japan; 11 Present Address: Division of Biomedical Information Analysis, Iwate Tohoku Medical Megabank Organization, Disaster Reconstruction Center, Iwate Medical University, Yahaba-cho, Shiwa-gun, Iwate 028-3694 Japan

## Abstract

Anaplastic lymphoma kinase (ALK) is an oncogenic receptor tyrosine kinase that is activated by gene amplification and mutation in neuroblastomas. ALK inhibitors can delay the progression of ALK-driven cancers, but are of limited use owing to ALK inhibitor resistance. Here, we show that resistance to ALK inhibitor in ALK-driven neuroblastomas can be attenuated by combination treatment with a p53 activator. Either ALK inhibition or p53 activator treatment induced cell cycle arrest, whereas combination treatment induced apoptosis, and prevented tumour relapse both in vitro and in vivo. This shift toward apoptosis, and away from cell-cycle arrest, in the presence of an ALK inhibitor and a p53 activator, is mediated by inhibition of the ALK–AKT–FOXO3a axis leading to a specific upregulation of SOX4. SOX4 cooperates with p53 to upregulate the pro-apoptotic protein PUMA. These data therefore suggest a novel combination therapy strategy for treating ALK-driven neuroblastomas.

Neuroblastoma (NB) is a common paediatric solid tumour, and is the cause of 9.1% of cancer-related deaths in children^1,2^. NB is classified into three groups according to The International Neuroblastoma Risk Group (INRG) classification system^3^. The high-risk group is characterised by amplification of the MYCN oncogene, and has a poor prognosis, with a 5-year survival rate of 40–50%. Although multimodal chemotherapeutic and immunotherapeutic strategies have improved survival in patients with high-risk disease, newer and better therapeutic strategies are still required^4^.

Anaplastic lymphoma kinase (ALK) belongs to the family of receptor tyrosine kinases (RTKs). The gene encoding ALK is the most frequently mutated gene in NB, and is a cause of familial NB^5–8^. Therefore, ALK inhibitors have the potential to be effective therapeutic agents for NBs harbouring ALK mutations. Additionally, they may be effective in NBs harbouring amplification of the *ALK* gene, which currently represent ~2% of total NB cases^9,10^. Most of the NBs with an amplification of ALK also harbour an amplification of MYCN, and have very poor prognosis^10^. Crizotinib, a first generation ALK inhibitor, has been studied in clinical trials for the treatment of paediatric cancer (NCT01606878)^11^. However, it had limited efficacy, due to primary resistance to crizotinib in NBs^10,12^. Alectinib is a second-generation ALK inhibitor, which overcomes the acquired resistance ascribed to gatekeeper mutations^13^. However, the efficacy of this second-generation ALK inhibitor in NBs harbouring ALK amplifications remains unclear. Resistance to ALK inhibitors has also become a critical issue in the treatment of non-small cell lung carcinoma (NSCLC) bearing the ALK-fusion gene^14,15^. Unfortunately, the mechanism by which NBs harbouring amplification of the *ALK* gene become resistant to ALK inhibitors is poorly understood.

The function of the tumour suppressor protein p53 is often found to be inactivated in various tumours, either through mutations or by perturbation of its regulatory pathways^16–18^. p53 is a transcription factor which, following activation in response to various cellular stresses, induces the expression of various p53-target genes (e.g., p21, PUMA, BAX, and NOXA) that act to prevent tumour development. Although p53 is frequently found to be mutated in various tumours, some types of tumours, including NB, retain wild-type p53; p53 status has been found to be associated with response to chemotherapy in some cases^19,20^. Therefore, activation of p53 is a promising therapeutic strategy for the treatment of various types of tumours^21^. The p53 protein is ubiquitinated by a variety of E3 ubiquitin ligases, including MDM2, and subsequently degraded through the ubiquitin–proteasomal system^18^. Nutlin-3a, and its derivative RG-7112, are p53 activators that can suppress the p53–MDM2 interaction, and lead to the stabilisation of p53 by preventing its proteasomal degradation^22,23^. These compounds activate the p53 pathway in cancer cells harbouring wild-type p53, but they have been shown to have limited efficacy in patients^21^. Although NBs almost always retain wild-type p53, the effect of p53 activators on resistance to ALK inhibitors in ALK-driven NBs remains unclear. In this study, we found that the p53 activators suppress the re-growth of ALK-driven NBs, which are resistant to ALK inhibitors, and reveal the mechanisms by which p53 activators enhance the efficacy of the ALK inhibitors.

## Results

### The ALK inhibitor induces cell-cycle arrest but not cell death in ALK-driven NB cells

**Fig. 1:**
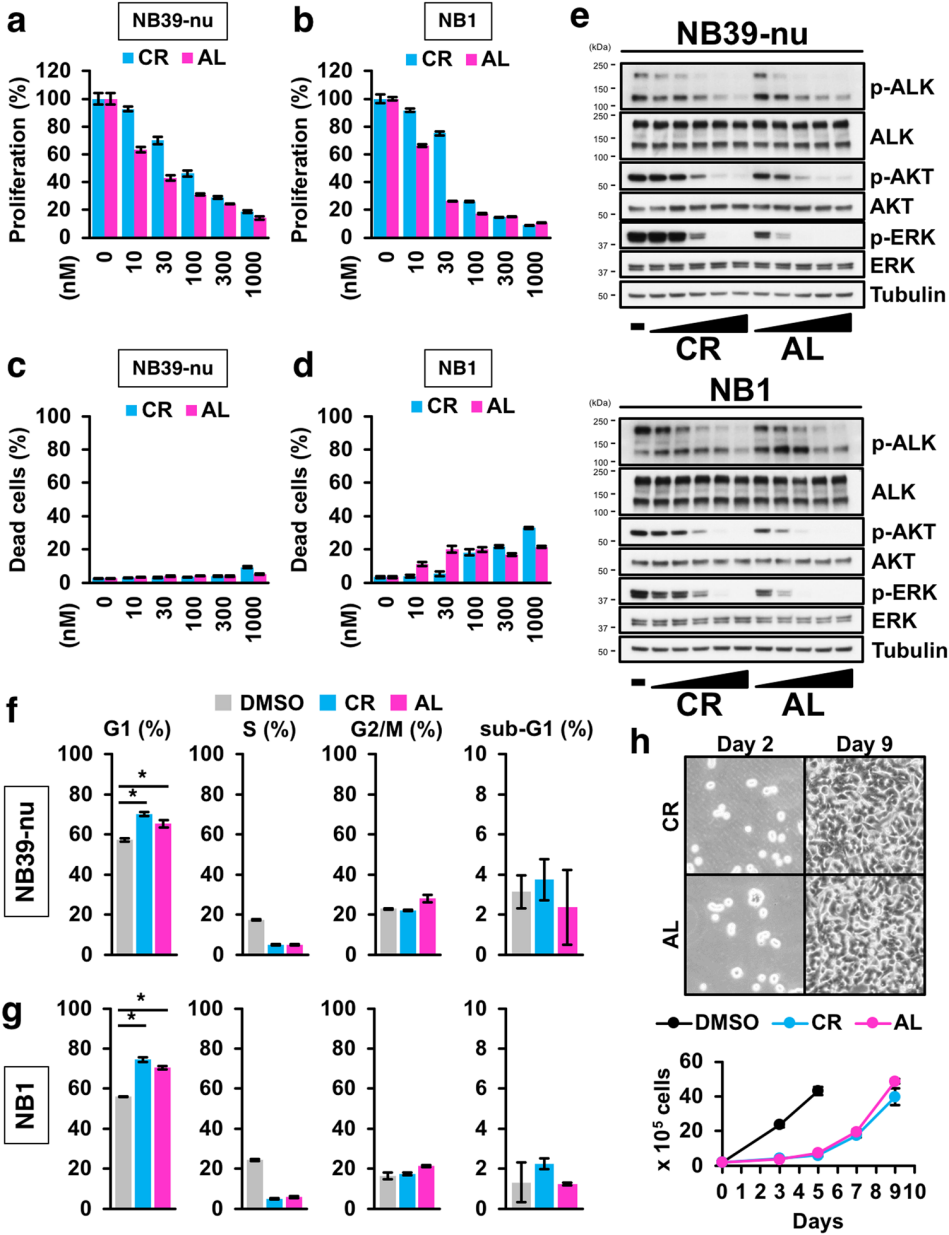
ALK inhibitors elicit cell-cycle arrest, but not cell death, in ALK-driven neuroblastomas. **a**,**b** Inhibition of cell proliferation in ALK-positive neuroblastoma cells by ALK inhibitors. NB39-nu cells (**a**) and NB1 cells (**b**) were treated with either crizotinib (CR) or alectinib (AL) for 48 h and a cell viability assay was performed. **c**,**d** Negligible or no induction of cell death in cells treated with ALK inhibitors. NB39-nu cells (**c**) and NB1 cells (**d**) were treated as in (**a**,**b**) and a CytoTox GLO assay was performed. **e** Response of the ALK-mediated pathway in ALK-driven NB cells to ALK inhibitors. NB39-nu cells or NB1 cells were treated with ALK inhibitors (10–1000 nM) for 6 h, as indicated. An immunoblotting analysis was conducted using the indicated antibodies. **f**,**g** A flow cytometry analysis of ALK-driven neuroblastoma cells treated with ALK inhibitors to assess cell-cycle stage. Data from NB39-nu (**f**) and NB1 (**g**) cells are shown. **h** Re-growth assay of NB39-nu cells following the withdrawal of ALK inhibitors. Detailed experimental procedures are described in the Methods section and Supplementary Figure 1e. All data are shown as the mean ± SD (*n* = 3), except for (**h**). In (**h**), the data are shown as the mean ± SEM (*n* = 4). The asterisk (*) signifies a *p* value < 0.05. All experiments were repeated at least three times

We first assessed the effect of two ALK inhibitors, crizotinib and alectinib, on cell viability in ALK-driven NBs including the ALK-amplified NB39-nu cell line, the ALK-amplified NB1 cell line, and the ALK-mutated SHSY5Y cell line. Both ALK inhibitors abrogated cell proliferation in the two ALK-driven NB cell lines, compared with IMR32 NB cells, which harbour a wild-type ALK (Fig. 1a, b; Supplementary Figure 1a, 1b). Intriguingly, although ALK inhibitors showed effective growth inhibition, the ALK inhibitors had little or no effect on cell death, as measured using the CytoTox GLO assay, which evaluates the number of dead cells, even at the highest dose of crizotinib and alectinib (Fig. 1c, d; Supplementary Figure 1c). Remarkably, this lack of effect of the ALK inhibitors on cell death occurred under conditions where the main signalling pathways (i.e. the AKT and ERK pathways) leading from ALK were completely inhibited, as shown by immunoblotting (Fig. 1e; Supplementary Figure 1d).

### Little or no induction of cell death by ALK inhibitors allows for the acquisition of drug-tolerance in NB cells

Given the result that ALK inhibitors appeared to induce cell-cycle arrest rather than cell death, we next analysed the effect of ALK inhibitors on inhibition of the cell-cycle in detail. A flow cytometry analysis revealed that both ALK inhibitors predominantly led to G1 arrest, but not cell death, even in ALK-driven NB, as assayed by measurement of the sub-G1 population (Fig. 1f, g). These data suggest that ALK inhibitors are incapable of inducing cell death. In the presence of either of the ALK inhibitors, ALK-driven NB cells were still viable, even though the ALK inhibitors suppressed ALK auto-phosphorylation and its downstream signalling pathways (Fig. 1c, d, e). Previous studies have shown that several different ALK mutations can be observed in NB (e.g. F1174L) corresponding with mutations involved in the acquired resistance of ALK-positive NSCLC^14,24^. In the case of our study, the two ALK inhibitors studied had little or no cytotoxic effect in ALK-mutated NB cells, as well as in ALK-amplified NB cells. Therefore, we hypothesised that the low cytotoxicity of the ALK inhibitors may be because of resistance to ALK inhibitors in NB cells. Consequently, we examined whether ALK-driven NB cells can re-grow after withdrawal of the ALK inhibitors. As a result, we found that withdrawal of the ALK inhibitors after 48 h of treatment allowed for re-growth of NB cells to occur (Fig. 1h; Supplementary Figure 1e, 1f, 1g), suggesting that the ALK inhibitors are unable to completely suppress tumour growth, and that a novel therapeutic strategy is therefore required for effective induction of cell death, but not cell-cycle arrest, in ALK-driven NB cells.

### Comprehensive expression analyses reveal that ALK inhibitors alter the expression of cell cycle-related genes

**Fig. 2:**
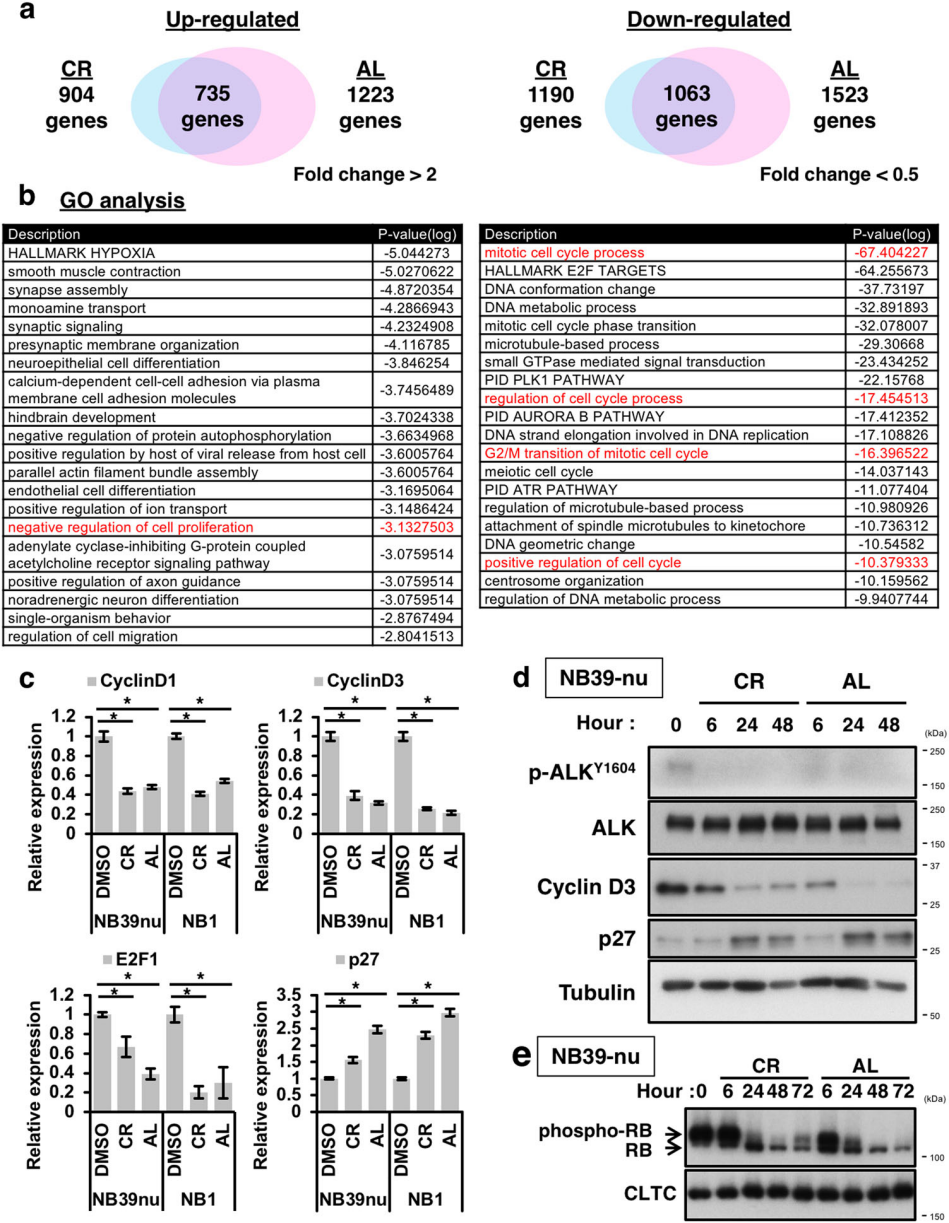
Microarray analysis reveals that ALK inhibitors alter the expression of cell cycle-related genes. **a** Summary of microarray analysis. NB39-nu cells were treated with 1000 nM of the two ALK inhibitors for 24 h, and were then subjected to a microarray analysis (see the Methods section). The numbers of genes upregulated, or downregulated, in common in NB39-nu cells treated with the two ALK inhibitors are shown as Venn diagrams. **b** Summary of Gene ontology (GO) analysis. A gene ontology analysis using Metascape (http://metascape.org/) was performed. The GO terms of the upregulated genes (left) and the downregulated genes (right) are shown. (See also supplementary Table 1). **c**,**d** Alterations in the expression of cell cycle-related genes in response to the two ALK inhibitors. NB39-nu or NB1 cells were treated with 1000 nM of the two ALK inhibitors for 24 h (**c**) or NB39-nu cells were treated as indicated (**d**) and qRT-PCR analysis (**c**) and an immunoblot analysis (**d**) were carried out. **e** The two ALK inhibitors induce dephosphorylation of pRb. NB39-nu cells were treated with either 1000 nM crizotinib (CR) or alectinib (AL) for the indicated times and subjected to immunoblot analysis. CLTC is the loading control. All quantitative data are shown as the mean ± SD (*n* = 3). *p < 0.05. All experiments were repeated at least three times

To elucidate the detailed mechanism by which ALK inhibition induces cell-cycle arrest, we performed comprehensive gene expression analyses using a DNA chip. From these analyses, common genes altered by both of the ALK inhibitors, crizotinib and alectinib, were identified and subjected to a gene ontology analysis using Metascape (Fig. 2a, b; Supplementary Table 1, see also the Methods section)^25^. The data showed that many cell cycle-related genes, as well as E2F1-target genes, were downregulated by ALK inhibition. In addition, many genes associated with the negative regulation of cell proliferation were upregulated (Fig. 2b). To further confirm these results, quantitative reverse-transcription PCR (qRT-PCR) and immunoblot analyses were carried out, and revealed that ALK inhibition principally downregulates the expression of Cyclin D1, Cyclin D3, E2F1, and up-regulates the expression of the cyclin-dependent-kinase inhibitor p27 (Fig. 2c, d). Furthermore, dephosphorylation of the phospho-RB protein, which leads to inactivation of E2F1, was also seen following ALK inhibition (Fig. 2e). Taken together, we conclude that the ALK inhibitors induce G1 arrest in ALK-driven NB cells by altering the expression of gene related to the progression from the G1 to the S phase in the cell cycle.

### ALK inhibitors synergise with p53 activators better than with chemotherapeutic agents

**Fig. 3:**
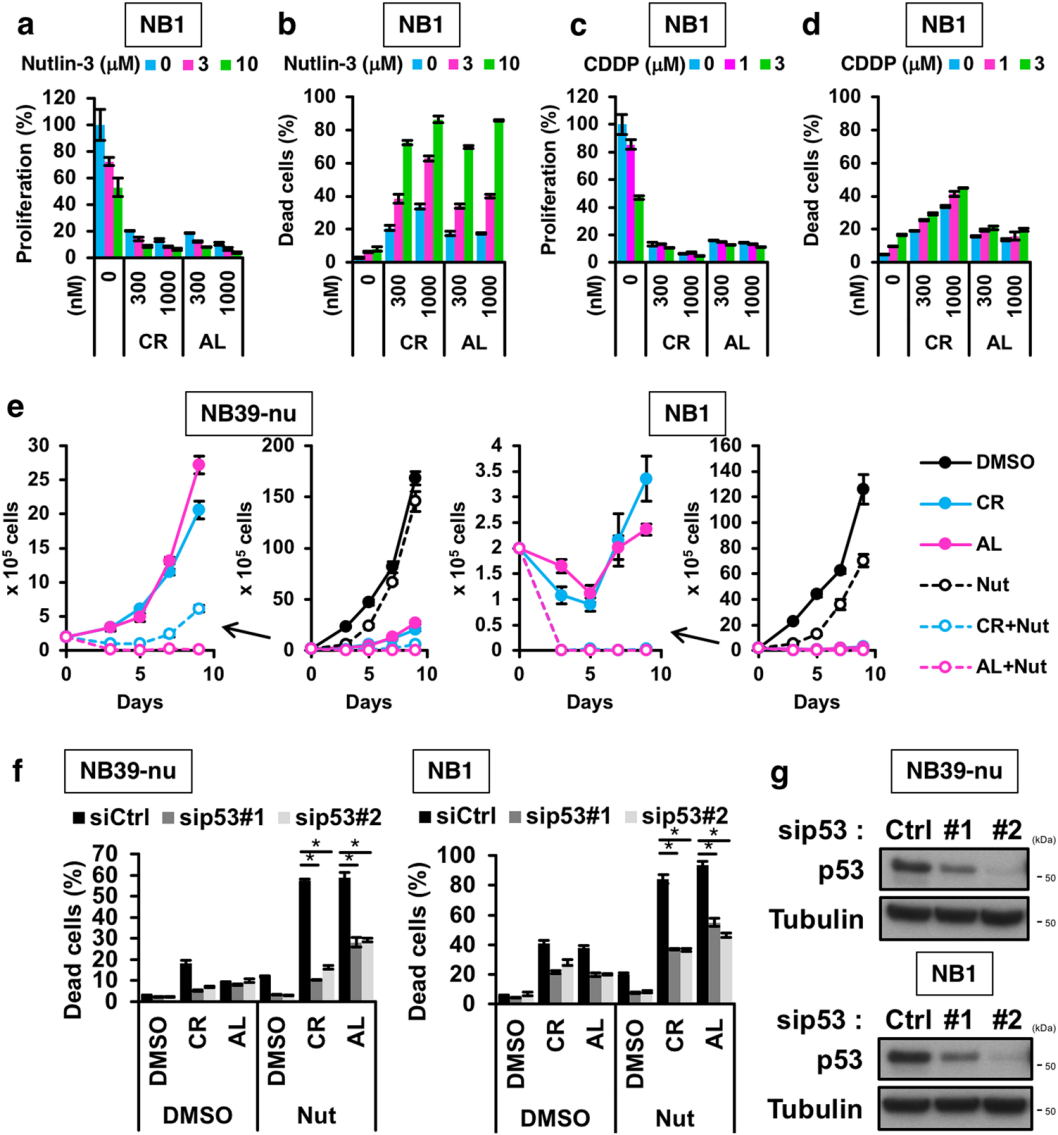
Combination of ALK inhibitor and p53 activator effectively induces cell death in vitro. **a**,**b** Combination treatment with ALK inhibitors and Nutlin-3 in NB1 cells. NB1 cells were treated with the indicated combinations of ALK inhibitors and Nutlin-3 for 48 h and then subjected to a cell viability assay (**a**) and a CytoTox GLO assay (**b**). **c**,**d** Combination treatment with ALK inhibitors and cisplatin in NB1 cells. NB1 cells were treated with the indicated combinations of ALK inhibitors and cisplatin (CDDP) for 48 h and then subjected to a cell viability assay (**c**) and a CytoTox GLO assay (**d**). **e** Inhibition of re-growth of ALK-driven neuroblastomas by combination treatment with the two ALK inhibitors and Nutlin-3. NB cells were treated with 1000 nM ALK inhibitor combined, or not, with 10 μM Nutlin-3 (Nut) for 48 h, and an in vitro re-growth assay was carried out. **f**,**g** Knockdown of p53 reverses the cell death elicited by the ALK inhibitor and Nutlin-3 combination treatment. NB cells were transfected with siRNA, then treated with 1000 nM of the two ALK inhibitors alone or in combination with 10 μM Nutlin-3 (Nut) for 48 h and subjected to a CytoTox GLO assay (**f**). The efficiency of p53 knockdown by two siRNAs in NB39-nu and NB cells were determined by immunoblot analysis (**g**). All data shown are the mean ± SD (*n* = 3) with the exception of (**e**). In (**e**), the result is shown as the mean ± SEM (*n* = 4). *p < 0.05. All experiments were repeated at least three times

**Fig. 4:**
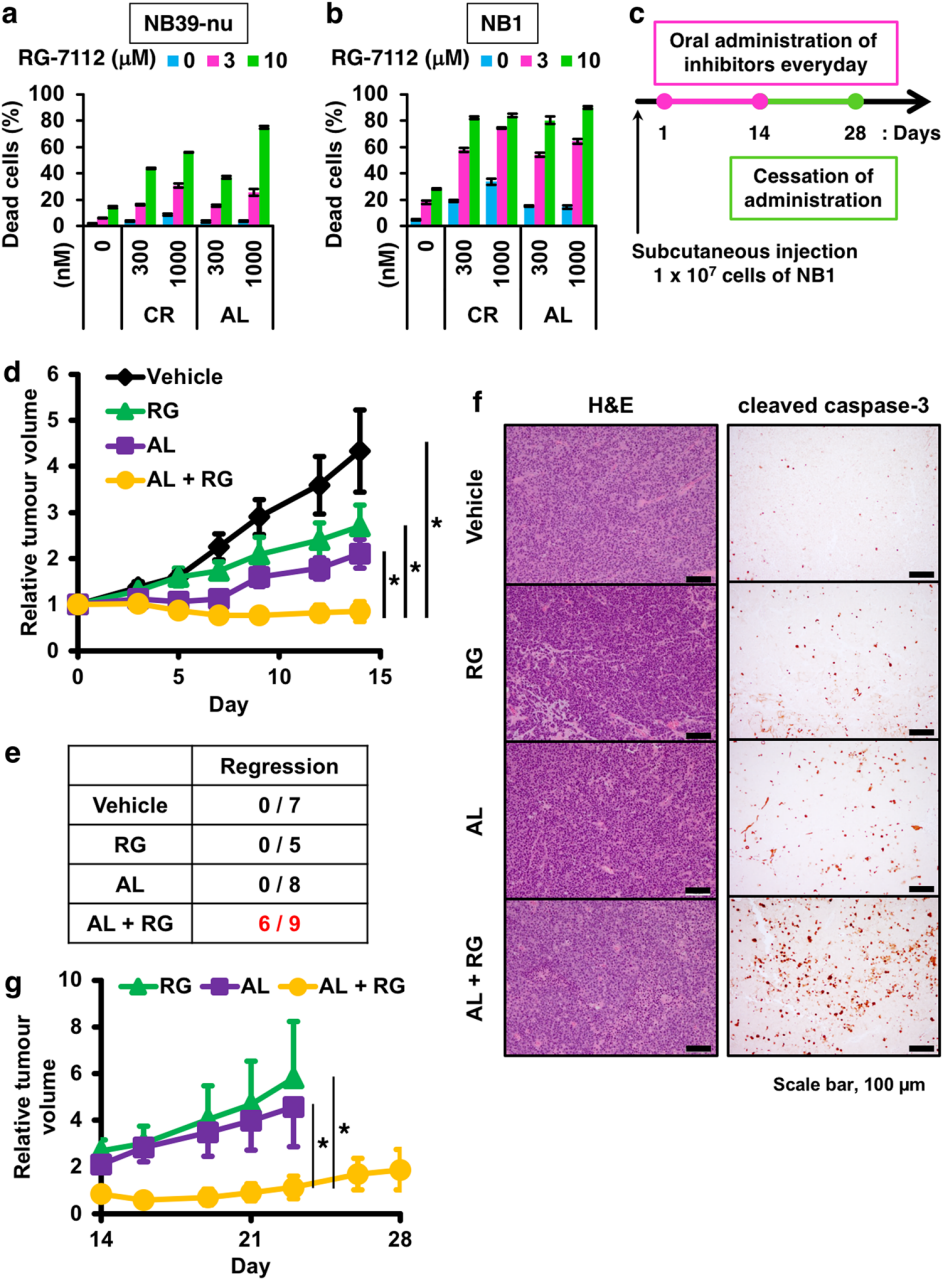
Combination treatment induces tumour regression and suppresses the relapse in vivo. **a**,**b** Induction of apoptosis by the combination of ALK inhibitors and the Nutlin-3 derivative RG7112 in ALK-driven neuroblastoma cells. NB39-nu cells (**a**) and NB1 cells (**b**) were treated with the indicated combinations of the ALK inhibitors and RG7112 for 48 h and subjected to a CytoTox GLO assay. The data are shown as mean ± SD (*n* = 3). These experiments were repeated at least three times. **c** Scheme used for the in vivo experiments. NB1 cells were injected subcutaneously into nude mice and drugs were orally administrated for 14 days every day after tumour formation (>90 mm^3^). Tumour volumes were measured three times a week. **d**, **e** Suppression of tumour growth by combination treatment with alectinib and RG7112. The relative tumour volumes are shown in graph (**d**). The data show the mean ± SEM *p < 0.05. The incidence of tumour regression (relative tumour volume at day 14 was lower than day 0) are summarised in **e**. **f** Detection of increased apoptosis in tumour sections from animals treated with the combination of alectinib and RG7112, as assayed by immuno-staining using an anti-cleaved caspase 3 antibody. For comparison, haematoxylin–eosin staining of the tumour sections are shown on the left. The scale bar indicates 100 µm. **g** Prevention of tumour relapse by combination treatment with alectinib and RG7112. The relative tumour volumes after ceasing oral administration of drugs are shown in the graph. The data shown are the mean ± SEM *p < 0.05 (Wilcoxon rank-sum test)

Recent reports have shown that the combined treatment of crizotinib with chemotherapy is more effective than single treatment in NBs harbouring aberrations on the ALK pathway^26^. In cancer cells harbouring wild-type p53, chemotherapy is accompanied by activation of the p53 pathway which leads to tumour suppression^20^. Therefore, we hypothesised that combining inhibition of the ALK pathway with activation of the p53 pathway may be an effective way to suppress the growth of ALK-driven NB cells. To validate our hypothesis, we first examined the effect of cisplatin, which leads to DNA double-strand breaks that activate the p53 pathway, on cell viability in ALK-driven NB cells, in the absence or presence of ALK inhibitors. As expected, cisplatin had a synergistic effect with the ALK inhibitors, as evaluated by a cell viability assay and a CytoTox GLO assay in ALK-amplified NB cells (Fig. 3c, d; Supplementary Figure 2c, 2d). However, genotoxic agents, such as cisplatin, can produce risks of adverse reactions and can cause chronic diseases in paediatric cancer patients^4^. To avoid this issue, we instead explored the use of Nutlin-3 and its derivative RG7112, both of which are inhibitors of the interaction between p53 and MDM2, a p53 E3 ubiquitin ligase. MDM2 inhibitors stabilise p53 by preventing its MDM2-mediated ubiquitin–proteasomal degradation. These p53 activators are therefore devoid of causing DNA damage^22,23^. Mutations in the *TP53* gene are rare in NB, and therefore the p53 pathway is intact in the NB cell lines used in this study^19,26,27^. Treatment of ALK-driven NB cells with Nutlin-3 induced cell-cycle arrest, but not cell death, similar to that seen with ALK inhibitors (Figs. 3a, b and 4a, b; Supplementary Figure 2e); in contrast, combination of the ALK inhibitors with either Nutlin-3 or RG7112 effectively induced cell death in ALK-driven NB cells (Figs. 3a, b and 4a, b; Supplementary Figure 2a, 2b).

We next assessed the combination index (CI) to evaluate whether these effects were synergistic or additional^28^. Measurement of CI for the combination of ALK inhibitors with Nutlin-3 showed a stronger degree of synergism than that for the combination with cisplatin, despite similar levels of p53 induction (Supplementary Figure 2f - k). Moreover, cell death induced by the combination treatment was dependent on the expression of p53, suggesting that the p53-mediated apoptotic pathway is required for cell death (Fig. 3f, g). We next examined whether the combination of ALK inhibitors with Nutlin-3 suppressed re-growth of the NB cells after removal of the inhibitors. In the presence of Nutlin-3, ALK inhibitors inhibited and delayed re-growth of the NB cells after removal of inhibitors, compared with ALK inhibitors or Nutlin-3 alone (Fig. 3e). Furthermore, we examined the cytotoxicity of the ALK inhibitors and the p53 activator Nutlin-3 in normal human lung fibroblast TIG-7 cells, in which the TP53 gene is intact^29,30^. As expected, ALK inhibitors did not inhibit cell growth in TIG-7 cells and Nutlin-3 induced cell-cycle arrest but not cell death (Supplementary Figure 3c, 3d). These data suggest that combined treatment of an ALK inhibitor with a p53 activator is a promising therapeutic strategy, devoid of side effects, for the treatment of ALK-amplified NB.

### Combination of alectinib with RG-7112 induces tumour regression in vivo

We next assessed whether the combined treatment is effective in vivo using a xenograft model. NB1 cells were subcutaneously implanted into nude mice and each inhibitor was administered in the implanted mice after tumour growth of NB1 cells for 14 days (Fig. 4c). Treatment with alectinib alone effectively suppressed tumour growth, as previously reported (Fig. 4d)^13^. Intriguingly, the combination treatment of alectinib with a Nutlin-3 derivative RG-7112 induced not only tumour suppression but also tumour regression in 6 of 9 mice; importantly, tumour regression was not observed with either single treatment alone (Fig. 4e). Moreover, the combination treatment suppressed the tumour relapse which could be seen at 2 weeks after the cessation of drug administration (Fig. 4g). Treatment with drugs had little or no effect on the body weight of mice, suggesting that there was no severe toxicity associated with the drug treatments (Supplementary Figure 3a). Immunohistochemistry and TUNEL staining showed that there was a huge induction of apoptosis in tumours treated with the combination of alectinib and RG-7112, suggesting that the combined treatment induces apoptosis more efficiently than the single treatment in vivo (Fig. 4f; Supplementary Figure 3b).

### The combination of ALK inhibitors with p53 activators induces cell death via the intrinsic apoptosis pathway

**Fig. 5:**
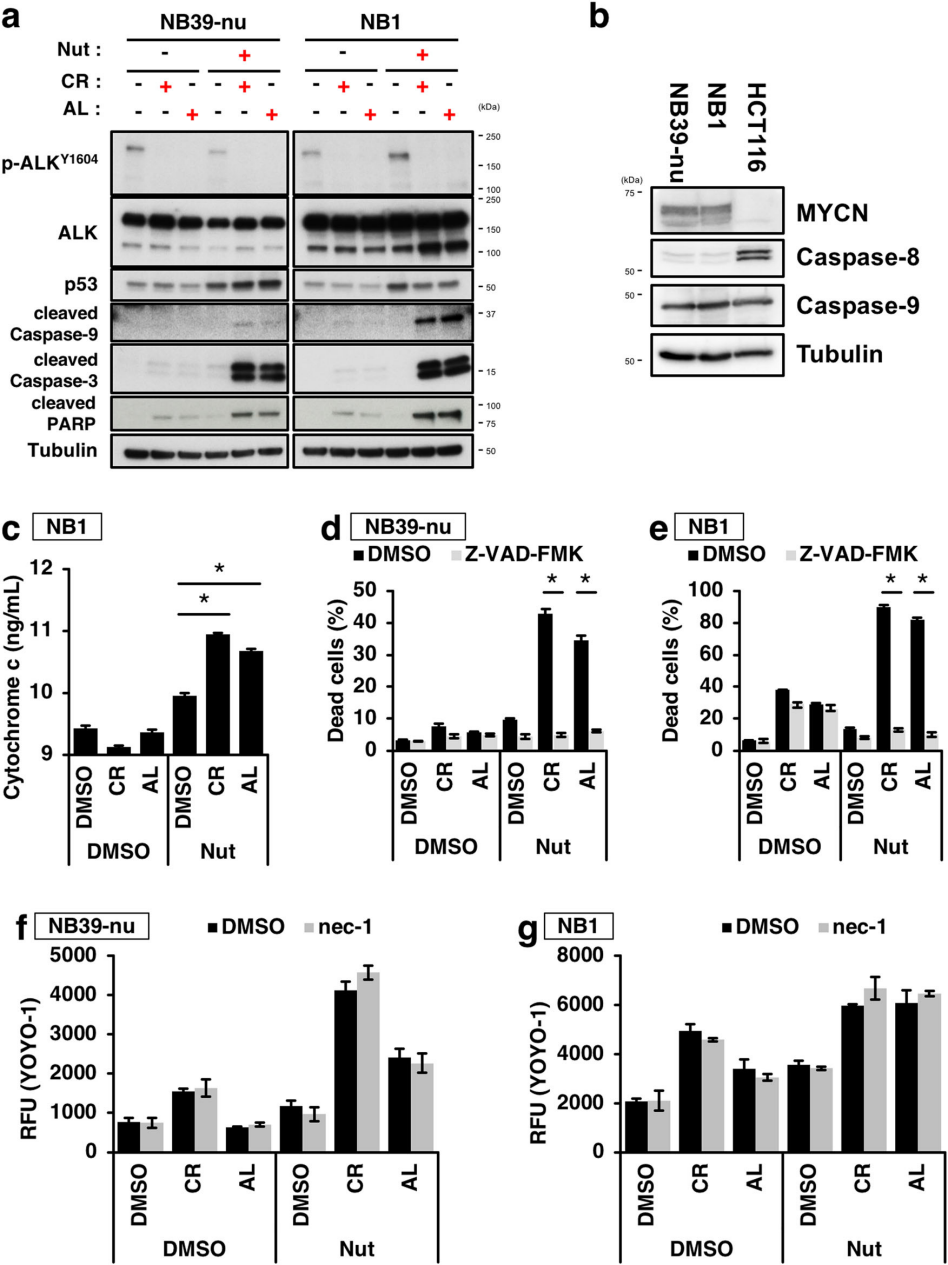
Combination treatment with the two ALK inhibitors and a p53 activator induces activation of the intrinsic apoptosis pathway. **a**,**b** Activation of mitochondria-mediated apoptotic pathway by combination treatment of the two ALK inhibitors with the p53 activator Nutlin-3. NB39-nu or NB1 cells were treated with 1000 nM of either of the two ALK inhibitors and 10 µM of Nutlin-3 for 16 h. The activation of the caspase cascade as a result of combination treatment was detected by immunoblot analysis (**a**). The expression levels of caspases-8 and -9 in NB39-nu or NB1 cells are shown in (**b**). **c** The increase in cytosolic cytochrome c following combination treatment. NB1 cells were treated with 1000 nM of either of the two ALK inhibitors and 10 µM of Nutlin-3 for 16 h. Cytosolic fractions were prepared by subcellular fractionation and the amount of cytochrome c present was quantified by ELISA. Data show the mean ± SEM (*n* = 3). *p < 0.05. **d**–**g** The caspase inhibitor Z-VAD-FMK, but not the RIPK1 inhibitor necrostatin-1, abolished the cell death elicited by the combination treatment. Z-VAD-FMK (100 µM) or necrostatin-1 (nec-1, 50 µM) were combined with the ALK inhibitors and Nutlin-3 as shown and a CytoTox GLO assay (**d**,**e**) or necroptosis assay (**f**,**g**) were performed. NB39-nu (**d**,**f**) and NB1 cells (**e**,**g**) were shown. Data indicate mean ± SD (*n* = 3). *p < 0.05. All experiments were repeated at least three times

p53 is a regulator of cell death that can act along the apoptotic and necroptotic pathways^31,32^. Therefore, we next investigated which type of cell death occurs as a consequence of combination treatment with an ALK inhibitor and a p53 activator. To achieve this, we first examined the activation of caspases and the efflux of cytochrome c from the mitochondria to assess apoptosis. Combination of either of the two ALK inhibitors with Nutlin-3 strongly induced the efflux of cytochrome c and activation of Caspase-9, which is known to be another marker of the intrinsic apoptosis pathway (Fig 5a,c); In contrast, activation of Caspase 8, which is known to be a marker of the extrinsic apoptosis pathway, was not detected in the ALK-amplified NB cell lines. It should be noted that the NB cell lines used in this study expressed relatively low levels of Caspase 8, confirming a previous report (Fig. 5b)^33^. We next addressed whether the cell death induced by the combination treatment is dependent on caspase activity. The pan-caspase inhibitor, Z-VAD effectively prevented the cell death caused by the combination treatment (Fig. 5d, e); in contrast, an inhibitor of necroptosis, necrostatin-1, had no effect on cell death (Fig. 5f, g)^34^. These data indicate that combination treatment with an ALK inhibitor and Nutlin-3 induces cell death through the intrinsic apoptotic pathway.

### Selective upregulation of PUMA is required for the induction of apoptosis elicited by the combination of an ALK inhibitor with a p53 activator

**Fig. 6:**
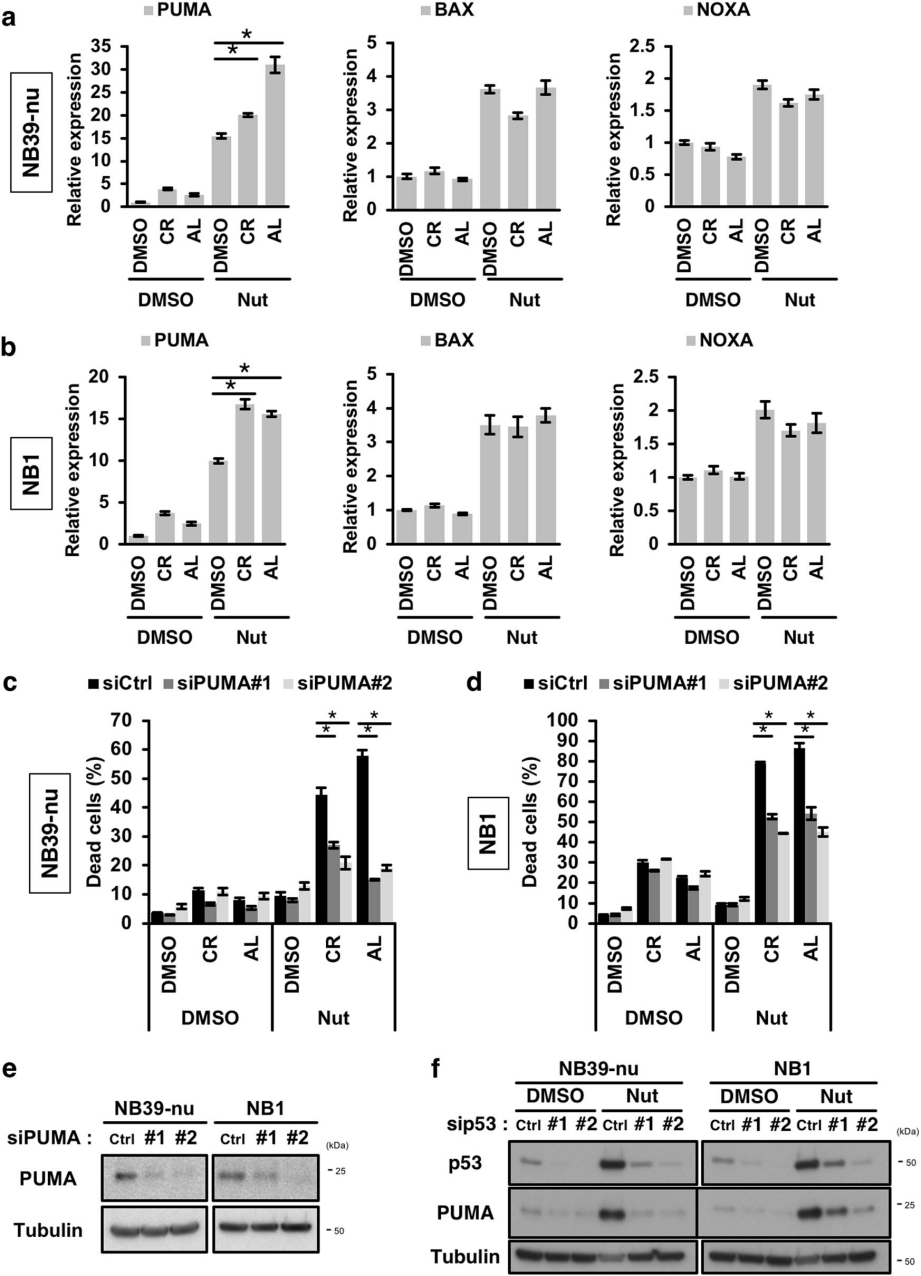
Selective upregulation of PUMA is important for cell death induced by combination treatment. **a**,**b** Induction of PUMA was potentiated by the combination of ALK inhibitors and Nutlin-3. NB39-nu cells (**a**) and NB1 cells (**b**) were treated with 1000 nM ALK inhibitor and 10 µM Nutlin-3 for 16 h. The expression of the indicated p53-target genes was determined by qRT-PCR analysis. **c**–**e** Knockdown of PUMA prevents the cell death elicited by combination treatment. NB cells were transfected with two siRNAs targeted to PUMA, then treated with 1000 nM ALK inhibitors and 10 µM Nutlin-3 for 48 h and subjected to a CytoTox GLO assay (**c**,**d**). The knockdown efficiency of PUMA by the two siRNAs was determined by immunoblot analysis (**e**). **f** Induction of PUMA is dependent on the expression of p53. NB cells were transfected siRNAs targeting p53 and then treated with 10 µM Nutlin-3 (Nut) for 16 h. An immunoblot analysis using the indicated antibodies is shown. The data show the mean ± SD (*n* = 3). *p < 0.05. All the experiments were repeated at least three times

To reveal the mechanism behind the synergistic effect between ALK inhibitors and p53 activators, we next explored the expression of p53-target genes, with a particular focus on genes belonging to the BCL2 family that are involved in the regulation of the intrinsic apoptotic pathway^35,36^. Unexpectedly, treatment with the ALK inhibitors enhanced the p53-mediated induction of PUMA (BBC3), a key player in p53-mediated apoptosis, although the induction of other pro-apoptotic BCL2 family members, such as BAX and NOXA (PMAIP1), was unaffected (Fig. 6a, b). We next addressed whether the expression of PUMA was required for the cell death induced by the combination of ALK inhibitors and Nutlin-3. Knockdown of PUMA prevented the apoptosis induced by the combination treatment, suggesting that the selective upregulation of PUMA is crucial for the induction of apoptosis elicited by the combination of an ALK inhibitor and Nutlin-3 (Fig. 6c, d, e). To investigate the p53 dependency, we examined the effect of p53 knockdown on PUMA expression. p53 knockdown attenuated the induction of PUMA elicited by treatment with Nutlin-3 (Fig. 6f). This result suggests that the induction of PUMA is dependent on the expression of p53 in ALK-amplified NB cells. We therefore hypothesised that factors regulated downstream of the ALK-mediated pathway must be present in cells, and that such factors may modulate p53-mediated PUMA expression. Various post-translational modifications (PTM) of p53 (e.g. phospho-Ser46, acetyl-Lys-373), and its binding partners (e.g. ASPPs), have been proposed as being factors responsible for the p53-mediated induction of apoptosis^35,37–39^. We next examined whether treatment with Nutlin-3, in the absence or presence of ALK inhibitors, affected PTMs of p53, or the expression of p53-binding partners. As shown in Supplementary Figure 4a, the levels of phosphorylation at Ser46, and acetylation at Lys373, were not affected by the combination of the ALK inhibitors and Nutlin-3. Using the expression data from the microarray study described above, we next analysed changes in the expression of p53-binding partners involved in p53-mediated apoptosis that were induced by treatment with ALK inhibitors; the genes are listed in Supplementary Figure 4b. From the list, SRY-related HMG-box 4 (SOX4) was identified as a candidate involved in upregulation of PUMA as a result of combination treatment.

### The expression of SOX4 is associated with prognosis in NB

A previous report has shown that a SOX4 consensus sequence is present in the promoter of the *BBC3* gene, and that SOX4 regulates the expression of PUMA (Supplementary Figure 4c)^40,41^. Moreover, SOX4 is known to directly interact with p53 and is involved in the induction of p53-target genes^42^. Therefore, we explored the relationship between the expression of SOX4 and prognosis in NB using the public database R2 (http://r2.amc.nl). A low level of expression of SOX4 was significantly associated with poor prognosis (Supplementary Figure 5a). We next examined whether the expression of SOX4 is affected by the status of amplification of MYCN, one of the main characteristics of high-risk NB. As shown in Supplementary Figure 5b, the expression of SOX4 was significantly decreased in MYCN-amplified NB compared with non-amplified NB. These data suggest that SOX4 acts as a tumour suppressor in NB.

### SOX4 is required for enhancement of the induction of PUMA expression and apoptosis elicited by the combination of an ALK inhibitor with a p53 activator

**Fig. 7:**
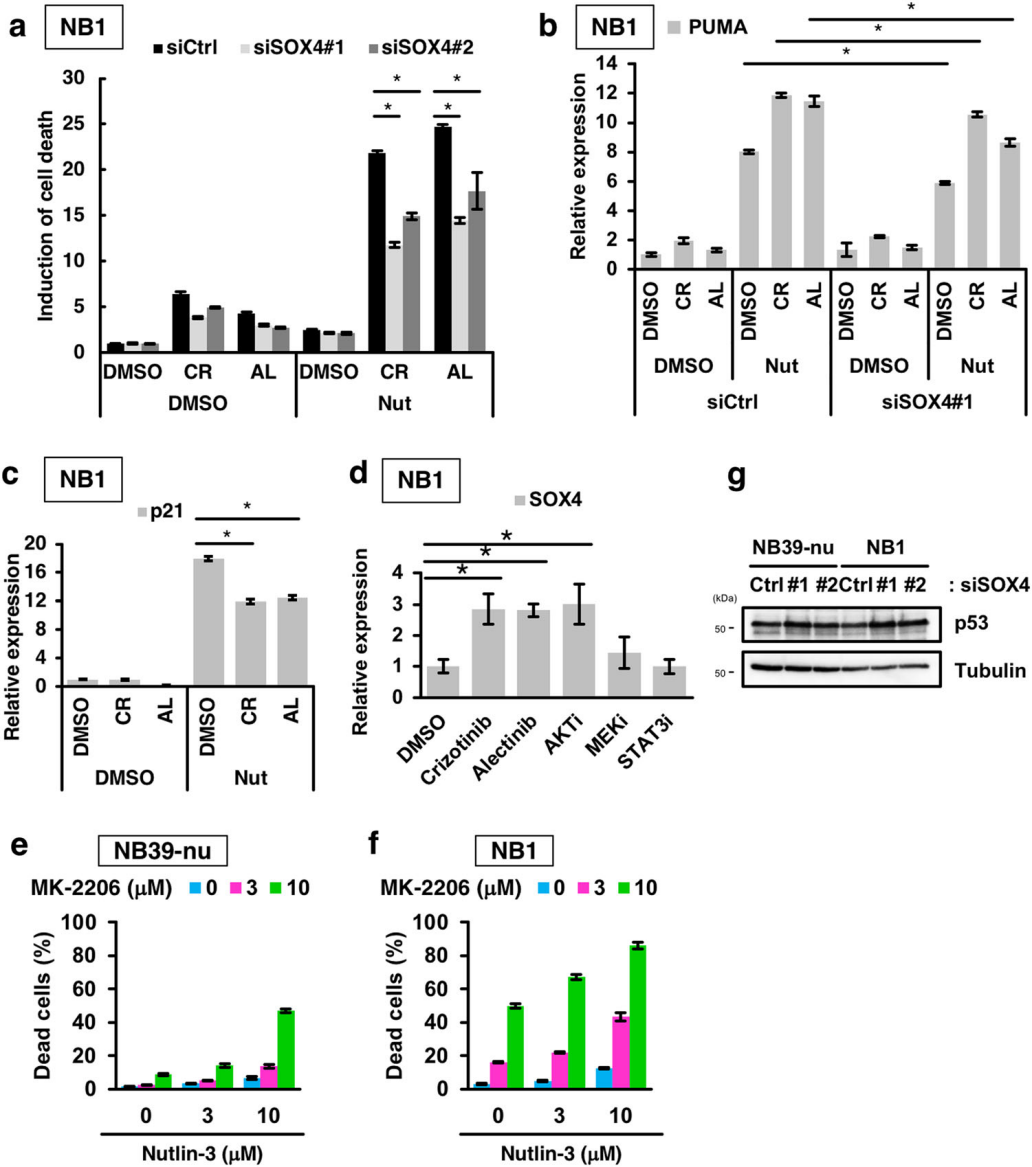
SOX4 is involved in the regulation of p53-mediated target selectivity and induction of cell death elicited by combination treatment. **a** Abrogation of cell death elicited by combination treatment following knockdown of SOX4. NB1 cells were transfected with either of the two SOX4 siRNAs, treated with 1000 nM ALK inhibitors, and 10 µM Nutlin-3 for 48 h, and a CytoTox GLO assay was carried out. **b**,**c** SOX4 is required for the specific induction of pro-apoptotic PUMA but not pro-cell cycle arrest p21. NB1 cells were transfected with a SOX4 siRNA and treated with 1000 nM ALK inhibitors and 10 µM Nutlin-3 for 16 h. The expression of PUMA and p21 were determined by qRT-PCR. See also Supplementary Figure 4e, 4f. **d** Regulation of SOX4 expression by the ALK–AKT axis. NB1 cells were treated with the indicated inhibitors (crizotinib, alectinib:1 µM, AKTi: 5 µM, MEKi: 1 µM, STAT3i: 75 µM) for 24 h. The expression of SOX4 was then measured by qRT-PCR. **e**,**f** Combination treatment of Nutlin-3 with the AKT inhibitor MK-2206 in ALK-amplified neuroblastomas. NB39-nu cells (**e**) and NB1 cells (**f**) were treated with MK-2206 and Nutlin-3 as indicated for 48 h and a CytoTox GLO assay was performed. **g** The knockdown of SOX4 has no effect on the stability of p53 proteins. NB39-nu and NB1 cells were transfected with a SOX4 siRNA. After 48 h of transfection, an immunoblot analysis was carried out using the indicated antibodies. All data show the mean ± SD (*n* = 3). *p < 0.05. All experiments were repeated at least three times

We first validated whether treatment with ALK inhibitors induces the expression of SOX4 using qRT-PCR. The expression of SOX4 was upregulated by treatment with ALK inhibitors in ALK-amplified NB1 cells, but not in ALK wild-type IMR32 cells (Fig. 7d; Supplementary Figure 4d). We next examined whether the expression of SOX4 is required for the induction of apoptosis elicited by the combination of an ALK inhibitor with a p53 activator. Knockdown of SOX4 partially prevented the apoptosis induced by the combination treatment (Fig. 7a; Supplementary Figure 4e). Moreover, the expression of SOX4 was required for the upregulation of PUMA induced by the combination treatment (Fig. 7b). These data suggest that SOX4 mediates the selective upregulation of PUMA, and that it is required for the enhanced apoptosis observed as a result of the combination treatment. Intriguingly, the induction of p21 expression was attenuated by combination treatment but it was not affected by SOX4 knockdown (Fig. 7c; Supplementary Figure 4f). These data suggest that selective p53-target gene expression switches from genes involved in cell-cycle arrest to genes involved in apoptosis.

We next investigated how the expression of SOX4 is regulated by ALK-mediated signalling. The major signalling pathways that lie downstream of ALK signalling are the AKT, MAPK and STAT3 pathways^43^. We, therefore, examined whether treatment with inhibitors of AKT, MEK or STAT3 induces the expression of SOX4, similar to ALK inhibitors. Among these inhibitors, inhibition of the AKT pathway, but not the MEK and STAT3 pathways, increased SOX4 expression (Fig. 7d). We next examined how AKT regulates the expression of SOX4, and focused on the transcription factor FOXO3a, because an association of FOXO3a with SOX4 expression has previously been reported^44^. It is known that FOXO3a is inactivated through its phosphorylation by AKT and subsequently becomes sequestered in the cytoplasm^45^. Treatment with the ALK inhibitors induced the dephosphorylation of FOXO3a and led to nuclear translocation of the de-phosphorylated FOXO3a in NB cells (Supplementary Figure 6a, 6b). Importantly, FOXO3a knockdown partially prevented the SOX4 expression induced by treatment with the ALK inhibitors (Supplementary Figure 6c, 6d). These data suggest that ALK regulates the expression of SOX4 through the ALK–AKT–FOXO3a regulatory pathway. Moreover, combination treatment with an AKT inhibitor and a p53 activator efficiently induced apoptosis, compared to single treatment alone (Fig. 7e, f). Thus, SOX4 expression is suppressed as a result of AKT signalling, and abrogation of the AKT pathway by the ALK inhibitor induces SOX4 expression-enhancing PUMA expression, leading to apoptosis.

Finally, we investigated whether ALK-fusion genes and other RTKs, including epidermal growth factor receptor (EGFR), that activate the AKT pathway also have an effect on the expression of SOX4. ALK-fusion genes have been observed in anaplastic large cell lymphomas (ALCLs) as a nucleophosmin 1 (NPM1)-to-ALK fusion gene, and in lung adenocarcinomas (lung ADCs) as an EML4-to-ALK fusion gene. Therefore, cell lines harbouring these two respective ALK fusion genes were treated with ALK inhibitors and the expression of SOX4 was quantified. Intriguingly, treatment with ALK inhibitors induced SOX4 expression in both the ALCL and lung ADC cell lines harbouring the two different ALK fusion genes (Supplementary Figure 6e). Moreover, the expression of SOX4 in lung ADCs harbouring the EGFR was also induced by inhibition of EGFR signalling, suggesting that combination of RTK inhibitors, including ALK inhibitors and EGFR inhibitors, along with a p53 activator could be widely applicable to treat many tumours harbouring aberration in RTKs including the EGFR in lung ADCs (Supplementary Figure 6e).

## Discussion

To date, ALK has been considered to be an attractive therapeutic target in tumours that contain an activated ALK. In fact, several groups have reported that ALK inhibitors can suppress the growth of NBs harbouring ALK aberrations. However, a detailed analysis of how ALK-driven NBs, especially ALK-amplified NBs, respond to ALK inhibitors, has not been reported. In this study, we show that, in ALK-amplified NBs, ALK inhibitors mainly induce cell-cycle arrest, but not cell death, and that ALK inhibitors in combination with a p53 activator can switch from cell-cycle arrest to apoptosis more effectively than the single drug treatments alone. Intriguingly, SOX4 is involved in this shift from cell-cycle arrest to apoptosis and promotes PUMA expression by collaborating with activated p53 to efficiently cause tumour regression. Moreover, the expression of SOX4 is suppressed by the ALK–AKT–FOXO3a signalling pathway and whether cell-cycle arrest or apoptosis is executed depends on the SOX4 expression level.

Although previous studies have shown that ALK inhibitors induce activation of the apoptotic pathway, the efficiency of apoptotic induction has not been described in detail. Our data demonstrate that the induction of apoptosis is limited even in the presence of high doses of inhibitors, although cell growth and phosphorylation of ALK and its downstream signals are inhibited by relatively low doses of inhibitors in ALK-amplified NB cells, compared with ALK-mutated NB cells. Presumably, single treatment is not sufficiently cytotoxic and in cell cycle-arrested tumours, this low level of cytotoxicity permits the accumulation of mutations and adaption to the therapy. Therefore, the stimulation of apoptosis, but not cell-cycle arrest, is crucial in preventing tumour relapse and the acquisition of drug resistance. In this regard, combination therapy is one strategy that has been adopted to ensure efficient tumour killing^46^. The activation of p53 is one potential method of inducing apoptosis in tumours retaining wild-type p53, including tumours such as NBs.

Most tyrosine kinase inhibitors face the acquisition of resistance, either by mutation, or by activation of a bypass pathway^47,48^. ALK inhibitors, but also other RTK inhibitors, may therefore be insufficient to induce irreversible growth arrest and consequently will be a subject of drug resistance and relapse. Therefore, before tumours acquire drug resistance, the stimulation of apoptosis in the tumour cell is important for an effective therapy to be achieved. From the data presented here, it appears that the efficacy of RTK inhibitors can be increased by combining with a p53 activator, and that such a combination treatment could be applicable for the treatment of other cancers harbouring aberrant *RTK* genes.

Why does treatment with a combination of an ALK inhibitor and a p53 activator induce apoptosis? One possible explanation is the selective activation by combination treatment, of PUMA, a *p53*-target gene, in tumours bearing aberrant ALK activation. This is an unexpected and interesting result in the field of p53 biology (Fig. 6a, b). PUMA is a key player in p53-mediated apoptosis, and accounts for the apoptosis seen following combination treatment with ALK inhibitors and a p53 activator. Mechanistically, SOX4 is upregulated as a result of the ALK inhibitor, and this SOX4 then interacts cooperatively with p53, upregulated by the p53 activators Nutlin-3 or RG-7112, leading to an increase in PUMA expression. In addition, it has been reported that SOX4 is a p53-binding partner and can stabilise p53^42^. However, in ALK-amplified NBs, SOX4 knockdown had little or no effect on the expression level of p53, suggesting that SOX4 is not the main molecule involved in p53 stabilisation in NB cells (Fig. 7g). Similarly, although it has been reported that SOX4 enhances the acetylation of p53 through stabilisation of the p53-p300/CBP interaction, acetylated p53 was not detected in NB cells treated with a combination of ALK inhibitors and Nutlin-3^42^. This discrepancy may be dependent on the cellular context between different types of tumours. Therefore, further analyses of the interplay between p53 and SOX4 will be required to elucidate how combination treatment enhances the expression of the *PUMA* gene in the future.

In addition to ALK inhibitors, treatment of the lung ADC cells with gefitinib, an EGFR inhibitor, also increased the expression of SOX4. We speculate that other RTK inhibitors, beyond gefitinib, could also synergise with p53 activators, because SOX4 is upregulated by inhibition of RTK signalling. p53 is found to be mutated in half of human cancers; in other words, half of human cancer retain wild-type p53. If combination treatment with a p53 activator and an RTK inhibitor is more effective than single treatments alone, this strategy could be widely applicable for the treatment of various human cancers in the clinical setting.

It has recently been reported that combination treatment with crizotinib and chemotherapy has a synergistic effect on the inhibition of cell proliferation, and that p53 is important for the induction of apoptosis in ALK-mutated NB cells to suppress tumour growth^26^. Another group has recently shown that a combination of ceritinib, an ALK inhibitor, and CGM097, a MDM2 inhibitor, showed increased anti-tumour activity, and attenuated resistance to the ALK inhibitor in ALK-mutated NB cells^49^. Here, we demonstrate that treatment with ALK inhibitors in the presence of Nutlin-3 has a greater synergistic effect than combination with chemotherapy. The reason why Nutlin-3 is more effective than chemotherapy cannot be explained by the level of p53 accumulated in cells. It is possible that the lower cytotoxicity of Nutlin-3, compared with cisplatin, might produce a stronger synergistic effect. Why does Nutlin-3 have a lower toxicity than cisplatin despite a similar induction of p53? One possible reason is that p53 induces various target genes and leads to cell-cycle arrest or apoptosis in context-dependent manner. The low cytotoxicity of a p53 activator is favourable in paediatric patients. In paediatric cancers, survivors often suffer from chronic diseases that arise from the treatment itself^4^. Although a safety assessment of alectinib and RG7112 will be important for the use of these drugs to treat paediatric patients, it is possible that a combination treatment with these drugs improves the quality of life for these patients. In our animal experiments, treatment with either alectinib, or RG7112, slightly delayed tumour growth, whereas combination treatment with alectinib and RG7112 completely suppressed tumour growth and induced tumour regression in a half of the tumours implanted in the mice, without any side effects such as decreases in body weights. Despite this favourable outcome, detailed analyses of pharmacokinetic properties as well as adverse reactions in mice will be needed before any clinical application.

In conclusion, we report a connection between ALK signalling and p53-mediated apoptosis, and propose a novel therapeutic strategy for the treatment of ALK-amplified NB. In addition, our study supports that both a cell viability assay based on cell proliferation, and a cytotoxicity assay based on cell death, are important for accurately assessing efficacy in cancer therapy. Furthermore, our findings suggest that for the broad spectra of other tumours that contain an activated RTK (e.g. the EGFR) and express wild-type p53, the use of an RTK inhibitor combined with a p53 activator may be applicable. Finally, our data demonstrate that single treatment of ALK-amplified NBs with either an ALK inhibitor or a p53 activator is insufficient to induce apoptosis, and that combination treatment with an ALK inhibitor and a p53 activator is required to overcome resistance to the ALK inhibitor and to suppress tumour relapse by activating the pro-apoptotic pathway as a result of SOX4-mediated PUMA upregulation (Supplementary Figure 7).

## Materials and methods

### Cell lines

The human neuroblastoma cell lines (NB1, NB39-nu, IMR32, and SH-SY5Y) were provided by A. Nakagawara, T. Ushijima, and N. Hattori. HCT116 cells were provided from B. Vogelstein. TIG-7 and PC3 cells were provided from JCRB (Japan). H3122 cells were provided from W. Pao. SUP-M2 cells were provided from DSMZ (Germany). NB1, NB39-nu, IMR32, SH-SY5Y, PC3, H3122 and SUP-M2 cells were cultured in RPMI-1640 medium (Nacalai Tesque, Japan) supplemented with 10% foetal bovine serum (GIBCO, USA) and 100 U/mL penicillin/streptomycin (Sigma-Aldrich, Germany) at 37 °C in a 5% CO_2_ atmosphere. HCT116 and TIG-7 cells were cultured in DMEM (high-glucose) medium (Nacalai Tesque, Japan) supplemented with 10% foetal bovine serum and 100 U/mL penicillin/streptomycin at 37 °C in a 5% CO_2_ atmosphere. All cell lines were tested for mycoplasma contamination.

### Reagents and antibodies

Crizotinib and alectinib (CH5424802) were purchased from Cell Signaling Technology (USA) and ChemScene (USA), respectively. Nutlin-3 and RG7112 were obtained from Cayman (USA) and APExBIO (China), respectively. Z-VAD-FMK was purchased from Peptide Institute (Japan). Adriamycin was purchased from Sigma-Aldrich (Germany). Anti-ALK (#3633, 1:10000), anti-phospho-ALK (Y1604) (#3341, 1:1000), anti-p44/42 MAPK (ERK1/2) (#4695, 1:1000), anti-phospho-p44/42 MAPK (ERK1/2) (T202, Y204) (#4370, 1:2000), anti-AKT (#4691, 1:1000), anti-phospho-AKT (S473) (#4060, 1:2000), anti-PUMA (#12450, 1:1000), anti-cleaved caspase 3 (#9661, 1:1000), anti-cleaved caspase-9 (#9501, 1:1000), anti-caspase-8 (#9746, 1:1000), anti-cleaved PARP (#5625, 1:1000), anti-MYCN (#9405, 1:1000), anti-FOXO3a (#12829, 1:1000), and anti-phospho-FOXO3a (S253) (#13129, 1:1000) antibodies were from Cell Signaling Technology. Anti-p21 (ab109520, 1:1000) and anti-acetyl-p53 (K373) (ab62376, 1:1000) antibodies were obtained from Abcam (UK) and anti-p53 (sc-126, 1:2000) antibody was purchased from Santa Cruz Biotechnology (USA). Anti-tubulin (T5168, 1:5000) and anti-p27 (610242, 1:1000) antibodies were obtained from Sigma and BD Transduction laboratory (USA), respectively. Anti-pRB (MK-15-3, 1:1000) and anti-cyclin D3 (MK0013-3S, 1:1000) antibodies were purchased from MBL (Japan). The anti-phospho-p53 (S46) (1:1000) antibody has been previously described^37^.

### Microarray analysis

For quality control, RNA purity and integrity were evaluated by measuring the ratio of its optical densities at 260 nm and 280 nm, and analysed using an Agilent 2100 Bioanalyzer (Agilent Technologies, Palo Alto, CA, USA). RNA labelling and hybridisation were performed using the Agilent One-Color Microarray-Based Gene Expression Analysis protocol (Agilent Technology, v6.5, 2010). Briefly, total RNAs (200 ng) from each sample were linearly amplified and labelled with Cy3-dCTP. The labelled cRNAs were then purified using an RNeasy Mini Kit (Qiagen, Germany). The concentration and specific activity of the labelled cRNAs (pmol Cy3/μg cRNA) were measured using a NanoDrop ND-1000 (ThermoFisher Scientific, USA). Labelled cRNAs (600 ng) were fragmented by adding 10× blocking agent (5 μL) and 25× fragmentation buffer (1 μL), and then heated at 60 °C for 30 min. Finally, 2× GE hybridisation buffer (25 μL) was added to dilute the labelled cRNA. Hybridisation solution (50 μL) was dispensed into the gasket slide and placed on the SurePrint G3 Human Microarray, 8×60 K (Agilent Technologies). The slides were then incubated for 17 h at 65 °C in an Agilent hybridisation oven, following which they were washed at room temperature according to the Agilent One-Color Microarray-Based Gene Expression Analysis protocol (Agilent Technology, v6.5, 2010). The hybridised array was immediately scanned with an Agilent Microarray Scanner (Agilent Technologies) Raw data were extracted using Agilent Feature Extraction Software (v11.0.1.1). The raw gene data were automatically summarised using the Agilent feature extraction protocol to generate a raw data text file, thus providing expression data for each gene probed on the array. Array probes that contained Flag A in samples were filtered out. Selected gProcessedSignal values were logarithm transformed and normalised using the quantile method. Statistical significance for gene expression changes was determined using the fold change. Hierarchical cluster analysis was performed using complete linkage and Euclidean distance as a measure of similarity. All data analysis and visualisation of differentially expressed genes were conducted using R 3.1.2 (www.r-project.org). A metascape analysis was performed using genes regulated in common using default settings (see also Supplementary Table 1).

### Quantitative reverse-transcription PCR

qRT-PCR was performed as previously described^30^. The Thunderbird Sybr qPCR Mix (Toyobo, Japan) was used according to the manufacturer’s recommendations. The sequences of all primers used in this study are shown in Supplementary Table 2.

### Immunoblot analysis

Immunoblot analysis was carried out as described previously with miner modifications^50,51^. Briefly, cell pellets were lysed with lysis buffer (50 mM Tris-HCl, pH 7.2, 250 mM NaCl, 2 mM MgCl_2_, 0.1 mM EDTA, 0.1 mM EGTA, 1% NP40) supplemented with 1 mM DTT, a protease-inhibitor cocktail (Roche, Switzerland) and a phosphatase-inhibitor cocktail (Roche). Insoluble components were removed by centrifugation at 20000 × *g* at 4 °C for 15 min before the addition of SDS sample buffer including 2 M urea. SDS samples were denatured at 60 °C for 30 min and samples containing 10–30 μg protein were separated using 4–20 or 7.5% (for the detection of pRb) SDS-polyacrylamide gels. Blots were visualised using X-ray films (GE Healthcare, USA) or a Fusion Solo S chemiluminescence imager (Vilber-Lourmat, Germany).

### RNA interference

Small-interfering RNAs (siRNAs) targeting the indicated genes (sip53 (100 pmol), siPUMA (100 pmol), and siSOX4 (25 pmol)) were transfected in cells (1 × 10^6^) using LipofectamineRNAiMAX (Life Technologies, USA). Twenty-four hours after transfection, the same siRNAs were re-transfected into the same cells to enhance the knockdown efficiency. Twenty hours after re-transfection, cells were re-seeded in the appropriate multi-well plate and used for qRT-PCR, cell viability, and cytotoxicity assays. Knockdown efficiency was determined at 48 h after the second transfection. The sequences of siRNAs used in this study are listed in Supplementary Table 3.

### Cell viability and cytotoxicity assays

For cell viability assay, cells (5 × 10^3^ for NB1; 5 × 10^3^ for NB39-nu; 1 × 10^4^ for SH-SY5Y; 3 × 10^4^ for IMR32 cells) were seeded in 96-well plates and incubated at 37 °C for 24 h in a CO_2_ incubator. Cells were treated with various concentrations of ALK inhibitors (crizotinib or alectinib) and/or p53 activators (Nutlin-3 or RG7112) and cell viability was measured using a cell counting kit-8 (Dojindo, Japan) 48 h after treatment with the ALK inhibitors and/or the p53 activators. Absorbance was measured at 450 nm using a microplate reader (Perkin-Elmer, USA). For the re-growth assay, 48 h after treatment with culture media including the ALK inhibitors or the p53 activators, the culture media was removed and the cells were further cultured for 7 to 9 days and the number of cells was counted every second day to assess in vitro re-growth.

For the cytotoxicity assay, cells (2.5 × 10^3^ for NB1; 2.5 × 10^3^ for NB39-nu; 5 × 10^3^ for SH-SY5Y cells) were seeded in a 96-well plate (half-area) and incubated at 37 °C for 24 h in a CO_2_ incubator. Cells were treated with various concentrations of the ALK inhibitors (crizotinib or alectinib) and/or p53 activators (Nutlin-3 or RG7112) and cytotoxicity was measured using the CytoTox GLO assay (Promega, USA), according to the manufacturer’s protocol. The percentage of dead cells was evaluated as the number of dead cells divided by the total number of cells. The combination index was calculated using CompuSyn (Compusyn, USA). For the necroptosis assay, cells (1 × 10^4^ for NB1; 1 × 10^4^ for NB39-nu cells) were seeded in a 96-well black solid plate and incubated at 37 °C for 24 h in a CO_2_ incubator. These cells were treated with the ALK inhibitors (1000 nM) and/or p53 activators (10 μM) plus YOYO-1 (100 nM) in the absence or presence of necrostatin-1 (nec-1, 50 µM), an inhibitor of RIPK1 that is required for the induction of necroptosis, and the fluorescence signal (ex. 485 nm/em. 520 nm) was measured. A cytochrome c ELISA Kit (Invitrogen, USA) was used to measure the release of cytochrome c from mitochondria, according to the manufacturer’s protocol.

To prepare the cytosolic fraction, cells were suspended in subcellular fractionation buffer (250 mM sucrose, 20 mM HEPES (pH 7.5), 10 mM NaCl, 1.5 mM MgCl_2_, 1 mM EDTA, 1 mM EGTA) and cell suspensions were homogenised by passage through a syringe with a 25-gauge needle. After centrifugation for 15 min at 20000 × *g*, the supernatants were collected and used for the cytochrome c release assay.

### Cell-cycle analysis

Cells were treated with ALK inhibitors at a final concentration of 1 μM and incubated at 37 °C for 24 h. The treated cells were fixed with 70% ethanol on ice for 1 h, and washed once with PBS (+). The cell pellets were suspended with PI staining buffer (PBS (+), 0.1% Triton X-100, 10 μg/mL propidium iodide, 100 μg/mL RNase A) and incubated at room temperature for 30 min. Cell cycle was analysed using an EC800 flow cytometer (Sony, Japan).

### Animal experiments

NB1 cells (1 × 10^7^) were mixed with matrigel (Corning, USA) and the mixture was injected subcutaneously into the flank of 7-week-old female BALB/c-nu/nu mice (Charles River, Japan). After tumour volumes reached 90 mm^3^, alectinib, RG7112, and/or vehicles were orally administrated to the mice once per day. Alectinib was dissolved in 15% polyethyleneglycol-400 (PEG-400), 15% 2-hydroxypropyl-ß-cyclodextrin (HPCD), 10% Cremophor EL, 10% dimethyl sulfoxide (DMSO), and 0.02 N HCl, whereas RG7112 was suspended in a solution composed of 1% hydroxypropyl cellulose and 0.1% Tween-80. The animal experiment protocols were approved by the Committee for Ethics in Animal Experimentation, and the experiments were conducted in accordance with the Guideline for Animal Experiments of the National Cancer Center.

### Immunohistochemistry

Tumours were fixed in 4% paraformaldehyde in PBS(−), paraffin-embedded, and cut into sections. After hydration, the slides were incubated in 10 mM citrate buffer (pH 6.0) for 20 min at 121 °C, washed with extensive water and incubated in 3% H_2_O_2_ for 10 min at room temperature. Blocking was carried out using 5% normal goat serum in TBST for 1 h at room temperature. The anti-cleaved caspase 3 antibody (1:200) was diluted in SignalStain Antibody Diluent (Cell Signaling Technology, #8112) and sections were incubated with primary antibody overnight at 4 °C. To detect signals, SignalStain Boost Detection Reagent (Cell Signaling Technology, #8114) was incubated with the sections for 30 min at room temperature, and the SignalStain DAB Substrate Kit (Cell Signaling Technology, #8059) was used as the enzymatic substrate. Following dehydration, the slides were mounted using Mount-Quick (Daido Sangyo, Japan). Haematoxylin and eosin staining was carried out using slides obtained from serial sections. For TUNEL, a MEBSTAIN Apoptosis TUNEL Kit Direct (MBL, Japan) was used according to the manufacturer’s recommendations.

### Immunocytochemistry

Cells were fixed with 4% paraformaldehyde in PBS (−) for 15 min at room temperature, then permeabilized and blocked with blocking buffer (1% BSA, 0.3% Triton X-100 in PBS(−)) for 1 h at room temperature. Anti-FOXO3a antibody (1:300) was incubated overnight at 4 °C and an anti-rabbit IgG antibody coupled with Alexa Fluor 488 was used for detection. DRAQ5 was used to counter-stain nuclei. Images were acquired using a Leica SP5 confocal microscopy system.

### Statistical analysis

Data are presented as the mean ± standard deviation (SD) or standard errors of the mean (SEM) of three or more independent experiments. Statistical analysis was performed using a two-sided Welch’s *t*-test, a Wilcoxon rank-sum test (for Fig. 4f), and a log-rank test (for Kaplan–Meier survival analysis) with a significance level of *p* < 0.05. No statistical methods were used for sample size estimation. No randomisation and blinding were used in the experiments.

### Data availability

Microarray data sets are available in the Gene Expression Omnibus (GEO). (accession GSE107354)

## Electronic supplementary material

Supplementary Figures

Supplementary Table 1

Supplementary Table 2

Supplementary Table 3

## References

[1] Maris JM, Hogarty MD, Bagatell R, Cohn SL (2007). Neuroblastoma. Lancet.

[2] Smith MA (2010). Outcomes for children and adolescents with cancer: challenges for the twenty-first century. J. Clin. Oncol..

[3] Cohn SL (2009). The International Neuroblastoma Risk Group (INRG) classification system: an INRG Task Force report. J. Clin. Oncol..

[4] Oeffinger KC (2006). Chronic health conditions in adult survivors of childhood cancer. N. Engl. J. Med..

[5] Chen Y (2008). Oncogenic mutations of ALK kinase in neuroblastoma. Nature.

[6] George RE (2008). Activating mutations in ALK provide a therapeutic target in neuroblastoma. Nature.

[7] Janoueix-Lerosey I (2008). Somatic and germline activating mutations of the ALK kinase receptor in neuroblastoma. Nature.

[8] Mosse YP (2008). Identification of ALK as a major familial neuroblastoma predisposition gene. Nature.

[9] Miyake I (2002). Activation of anaplastic lymphoma kinase is responsible for hyperphosphorylation of ShcC in neuroblastoma cell lines. Oncogene.

[10] Bresler SC (2014). ALK mutations confer differential oncogenic activation and sensitivity to ALK inhibition therapy in neuroblastoma. Cancer Cell..

[11] Mosse YP (2013). Safety and activity of crizotinib for paediatric patients with refractory solid tumours or anaplastic large-cell lymphoma: a Children’s Oncology Group phase 1 consortium study. Lancet Oncol..

[12] Bresler SC (2011). Differential inhibitor sensitivity of anaplastic lymphoma kinase variants found in neuroblastoma. Sci. Transl. Med..

[13] Sakamoto H (2011). CH5424802, a selective ALK inhibitor capable of blocking the resistant gatekeeper mutant. Cancer Cell.

[14] Katayama R, Lovly CM, Shaw AT (2015). Therapeutic targeting of anaplastic lymphoma kinase in lung cancer: a paradigm for precision cancer medicine. Clin. Cancer Res..

[15] Toyokawa G, Seto T (2015). Updated evidence on the mechanisms of resistance to ALK inhibitors and strategies to overcome such resistance: clinical and preclinical data. Oncol. Res Treat..

[16] Olivier M, Hollstein M, Hainaut P (2010). TP53 mutations in human cancers: origins, consequences, and clinical use. Cold Spring Harb. Perspect. Biol..

[17] Petitjean A, Achatz MI, Borresen-Dale AL, Hainaut P, Olivier M (2007). TP53 mutations in human cancers: functional selection and impact on cancer prognosis and outcomes. Oncogene.

[18] Toledo F, Wahl GM (2006). Regulating the p53 pathway: in vitro hypotheses, in vivo veritas. Nat. Rev. Cancer.

[19] Vogan K (1993). Absence of p53 gene mutations in primary neuroblastomas. Cancer Res..

[20] Lu C, El-Deiry WS (2009). Targeting p53 for enhanced radio- and chemo-sensitivity. Apoptosis.

[21] Khoo KH, Verma CS, Lane DP (2014). Drugging the p53 pathway: understanding the route to clinical efficacy. Nat. Rev. Drug. Discov..

[22] Vassilev LT (2004). In vivo activation of the p53 pathway by small-molecule antagonists of MDM2. Science.

[23] Tovar C (2013). MDM2 small-molecule antagonist RG7112 activates p53 signaling and regresses human tumors in preclinical cancer models. Cancer Res..

[24] Sasaki T (2010). The neuroblastoma-associated F1174L ALK mutation causes resistance to an ALK kinase inhibitor in ALK-translocated cancers. Cancer Res..

[25] Tripathi S (2015). Meta- and orthogonal integration of influenza “OMICs” data defines a role for UBR4 in virus budding. Cell Host Microbe.

[26] Krytska K (2016). Crizotinib synergizes with chemotherapy in preclinical models of neuroblastoma. Clin. Cancer Res..

[27] Niizuma H (2006). Bcl-2 is a key regulator for the retinoic acid-induced apoptotic cell death in neuroblastoma. Oncogene.

[28] Chou TC (2010). Drug combination studies and their synergy quantification using the Chou-Talalay method. Cancer Res..

[29] Yamamoto K (1991). A new human male diploid cell strain, TIG-7: its age-related changes and comparison with a matched female TIG-1 cell strain. Exp. Gerontol..

[30] Otomo R (2014). TSPAN12 is a critical factor for cancer-fibroblast cell contact-mediated cancer invasion. Proc. Natl Acad. Sci. USA.

[31] Tu HC (2009). The p53-cathepsin axis cooperates with ROS to activate programmed necrotic death upon DNA damage. Proc. Natl Acad. Sci. USA.

[32] Vaseva AV (2012). p53 opens the mitochondrial permeability transition pore to trigger necrosis. Cell.

[33] Teitz T (2000). Caspase 8 is deleted or silenced preferentially in childhood neuroblastomas with amplification of MYCN. Nat. Med..

[34] Degterev A (2005). Chemical inhibitor of nonapoptotic cell death with therapeutic potential for ischemic brain injury. Nat. Chem. Biol..

[35] Beckerman R, Prives C (2010). Transcriptional regulation byp53. Cold Spring Harb. Perspect. Biol..

[36] Jeffers JR (2003). Puma is an essential mediator of p53-dependent and -independent apoptotic pathways. Cancer Cell..

[37] Oda K (2000). p53AIP1, a potential mediator of p53-dependent apoptosis, and its regulation by Ser-46-phosphorylated p53. Cell.

[38] Samuels-Lev Y (2001). ASPP proteins specifically stimulate the apoptotic function of p53. Mol. Cell.

[39] Sullivan A, Lu X (2007). ASPP: a new family of oncogenes and tumour suppressor genes. Br. J. Cancer.

[40] Jang SM (2013). Transcription factor Sox4 is required for PUMA-mediated apoptosis induced by histone deacetylase inhibitor, TSA. Biochem. Biophys. Res. Commun..

[41] Liu P (2006). Sex-determining region Y box 4 is a transforming oncogene in human prostate cancer cells. Cancer Res..

[42] Pan X (2009). Induction of SOX4 by DNA damage is critical for p53 stabilization and function. Proc. Natl Acad. Sci. USA.

[43] Hallberg B, Palmer RH (2013). Mechanistic insight into ALK receptor tyrosine kinase in human cancer biology. Nat. Rev. Cancer.

[44] Wang L, Brugge JS, Janes KA (2011). Intersection of FOXO- and RUNX1-mediated gene expression programs in single breast epithelial cells during morphogenesis and tumor progression. Proc. Natl Acad. Sci. USA.

[45] Tzivion G, Dobson M, Ramakrishnan G (2011). FoxO transcription factors; Regulation by AKT and 14-3-3 proteins. Biochim. Biophys. Acta.

[46] Cheok CF, Lane DP (2012). Seeking synergy in p53 transcriptional activation for cancer therapy. Discov. Med..

[47] Holohan C, Van Schaeybroeck S, Longley DB, Johnston PG (2013). Cancer drug resistance: an evolving paradigm. Nat. Rev. Cancer.

[48] Camidge DR, Pao W, Sequist LV (2014). Acquired resistance to TKIs in solid tumours: learning from lung cancer. Nat. Rev. Clin. Oncol..

[49] Wang, H. Q. et al. Combined ALK and MDM2 inhibition increases antitumor activity and overcomes resistance in human ALK mutant neuroblastoma cell lines and xenograft models. *Elife* **6**, 17137 (2017). 10.7554/eLife.17137 PMC5435462 28425916

[50] Ohata H (2013). NuMA is required for the selective induction of p53 target genes. Mol. Cell. Biol..

[51] Otsubo C (2014). TSPAN2 is involved in cell invasion and motility during lung cancer progression. Cell Rep..

