# Supplementary material for: The p53 activator overcomes resistance to ALK inhibitors by regulating p53-target selectivity in ALK-driven neuroblastomas
Source: Cell Death Discov. 2018 May 10;4:56. doi: 10.1038/s41420-018-0059-0 (PMC5945735; doi:10.1038/s41420-018-0059-0)
Supplement: Supplementary file 1 — Supplementary Figures [file 41420_2018_59_MOESM1_ESM.docx]

##
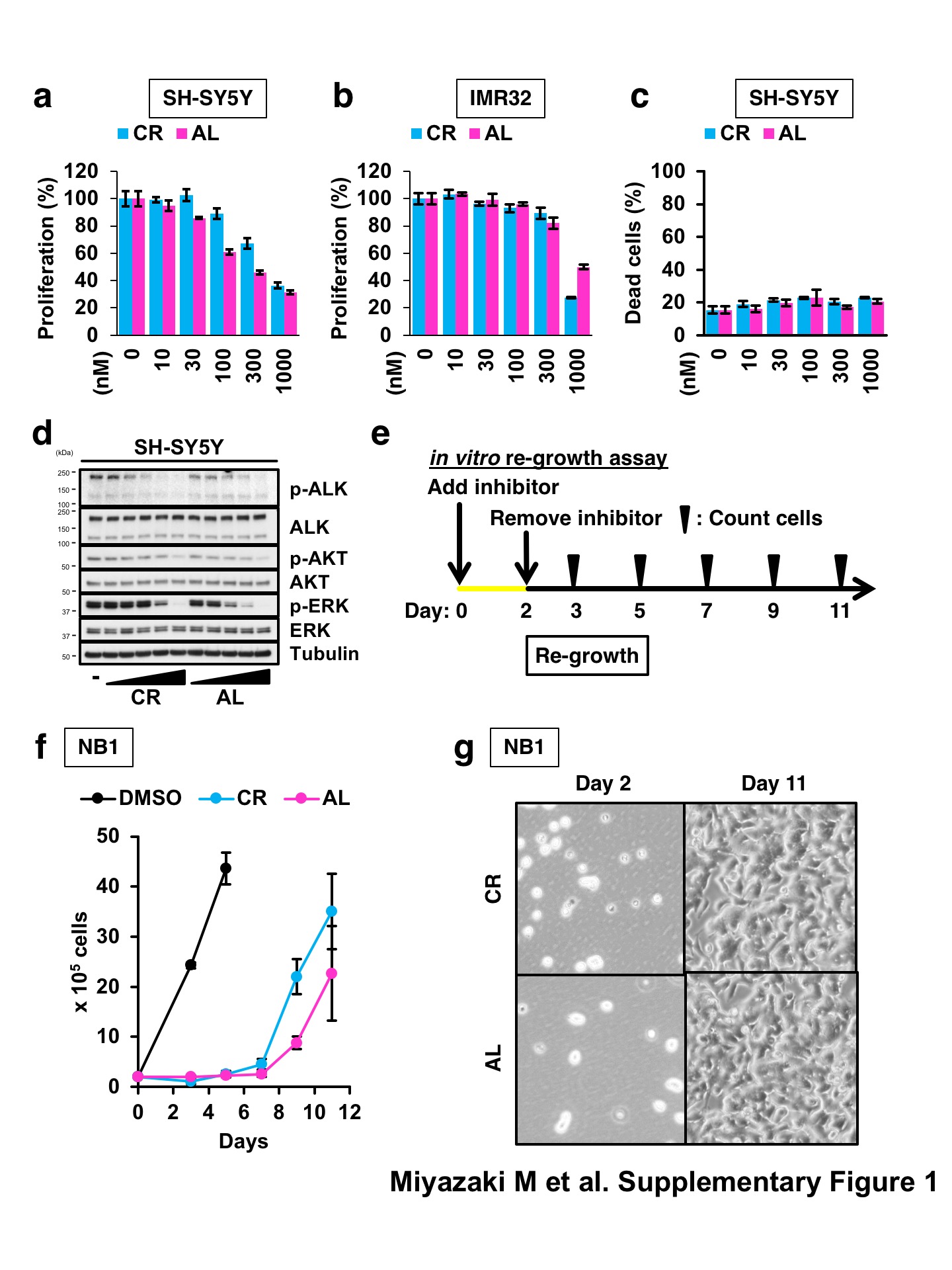


## **Supplementary Figure 1. ALK inhibitors elicit cell-cycle arrest rather than cell death in ALK-driven neuroblastoma cells and cells re-grow when the ALK inhibitors are removed (Related to Figure 1).**

**a, b,** Inhibition of cell proliferation in ALK-mutated (SH-SY5Y) and wild-type (IMR32) cells by the two ALK inhibitors. SY-SY5Y cells (a) and IMR32 cells (b) were treated with either crizotinib (CR) or alectinib (AL) for 48 h and a cell viability assay was performed. **c,** No induction of cell death in SH-SY5Y cells following treatment with the ALK inhibitors. SH-SY5Y cells were treated as indicated for 48 h and a CytoTox GLO assay was carried out. **d,** Response of the ALK-mediated signalling pathway in SH-SY5Y cells to the two ALK inhibitors. SH-SY5Y cells were treated with increasing concentrations of ALK inhibitors (10–1000 nM) for 6 h, as indicated. An immunoblot analysis using the indicated antibodies is shown. **e - g,** The scheme used for the *in vitro* re-growth assay is shown (e). Schematic of the *in vitro* re-growth assay procedure in NB1 cells. Cells were treated with either of the two ALK inhibitors for 48 hours, after which the inhibitors were removed and the number of cells were counted every two days from day 3 onwards. The number of cells (f) and images of the cells (g) are shown. All data are shown as the mean +/- SD (n = 3) except for (f) which is expressed as mean +/- SEM (n = 4). All experiments were repeated at least three times.

##
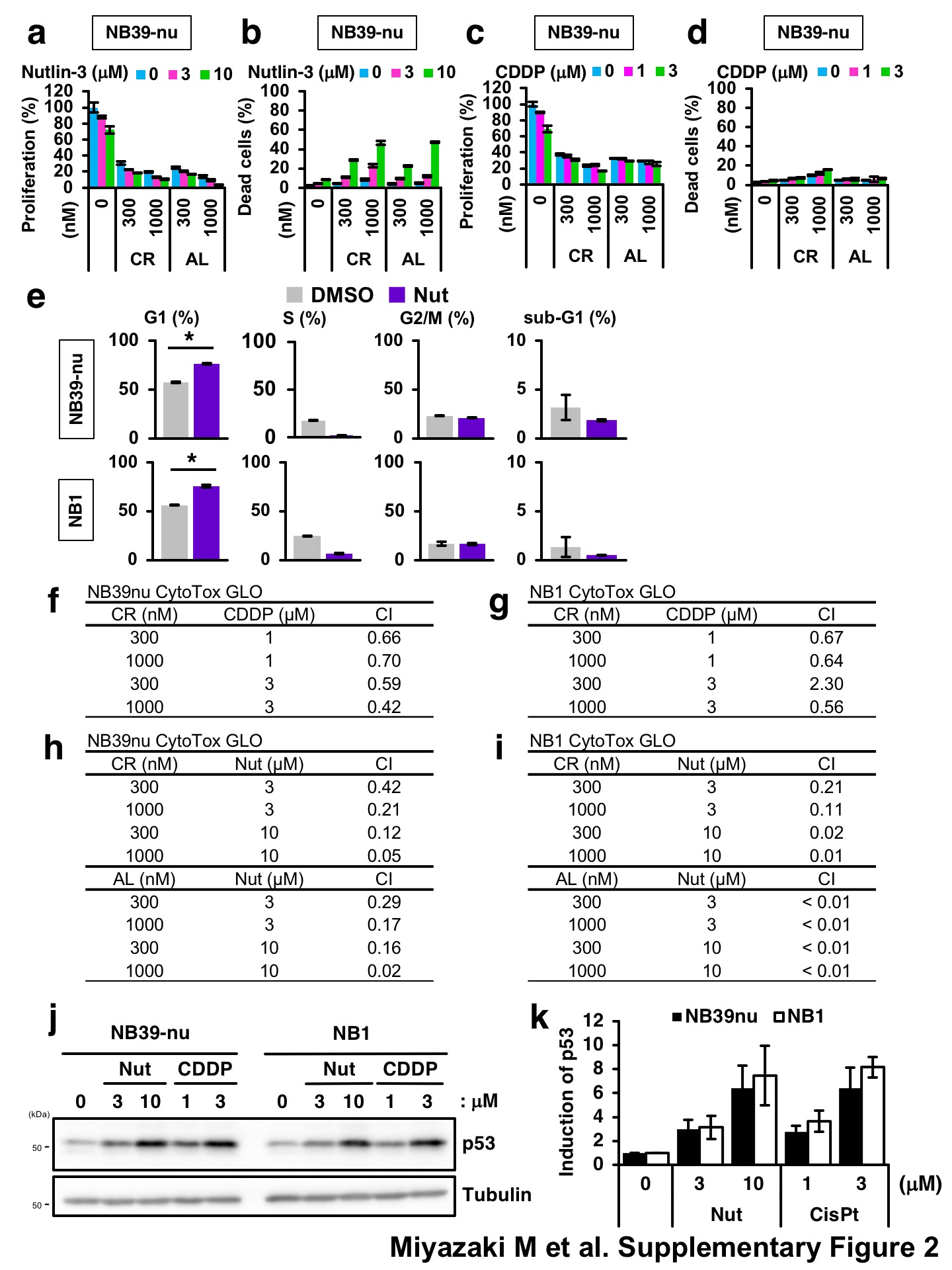


## **Supplementary Figure 2. Treatment with the ALK inhibitors in combination with a p53 activator is more potent than in combination with cisplatin (Related to Figure 3).**

**a - d,** Inhibition of cell-proliferation (a, c) and induction of cell death (b, d) following treatment with the two ALK inhibitors in combination with Nutlin-3 (a, b) or cisplatin (CDDP) (c, d) in NB39-nu cells. NB39-nu cells were treated as indicated for 48 h and a cell viability assay and a CytoTox GLO assay were carried out. Data are shown as mean +/- SD (n = 3). *p < 0.05. **e,** Flow cytometry analysis of ALK-driven neuroblastoma cells treated with Nutlin-3. NB1 or MB39-nu cells were treated with 10 µM Nutlin-3 and a cell-cycle analysis was performed. The data are shown as mean +/- SD (n = 3). *p < 0.05. **f - i,** Summary of the Combination Index (CI). The CI was calculated from all of the available CytoTox GLO assay using Compusyn software. The CI values the ALK inhibitor crizotinib (CR) with Cisplatin (CDDP) (f and g) or, crizotinib (CR) or alectinib (AL) with Nutlin-3 (Nut) (h and i), on NB39-nu cells (f and h) or NB1 cells (g and i) are shown. **j, k,** Comparison of the amounts of p53 induced by Nutlin-3 and cisplatin using immunoblotting (j) and quantification using densitometry (k). NB cells were treated as indicated for 16 h. An immunoblot analysis was conducted using the indicated antibodies. Data are shown as mean +/- SD (n = 3). *p < 0.05. All experiments were repeated at least three times.

##
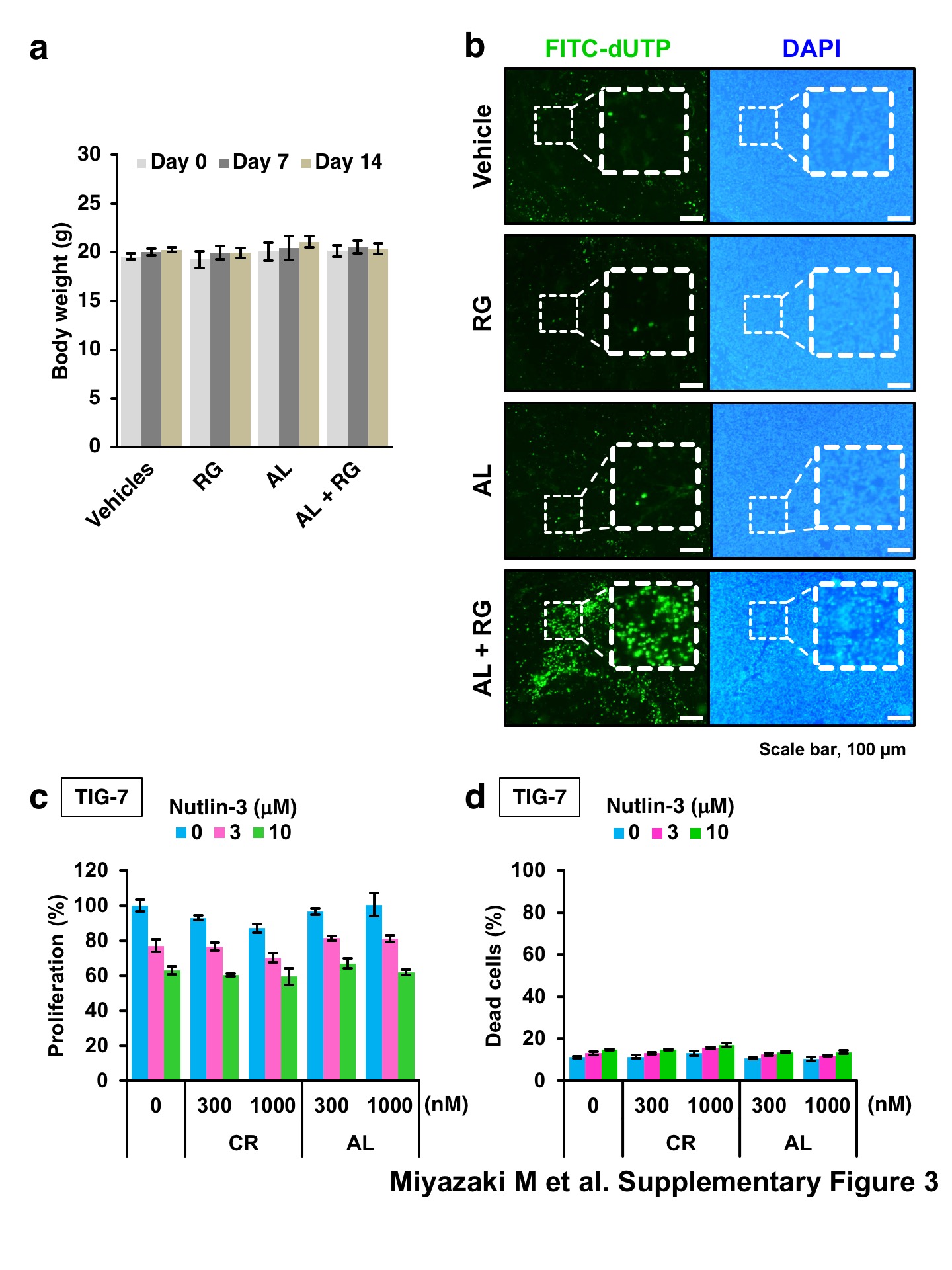


## **Supplementary Figure 3. Combination treatment of alectinib and RG7112 induces apoptosis without remarkable adverse effects *in vivo* (Related to Figure 4)**

**a,** Body weight of mice assessed over 14 days of treatment. The data are mean +/- SEM. **b,** Detection of increased apoptosis in the tumour sections by combination treatment, as assayed by TUNEL staining. Mice were sacrificed and tumours were resected at day 14. The scale bar indicates 100 µm. **c, d,** Combinational effect of the two ALK inhibitors with the p53 activator Nutlin-3 on normal fibroblast cells. The normal human fibroblast cells, TIG-7, were treated as indicated for 48 h and a cell viability assay (c) and a CytoTox GLO assay (d) were carried out. Data show the mean +/- SD (n = 3). These experiments were repeated at least three times.

##
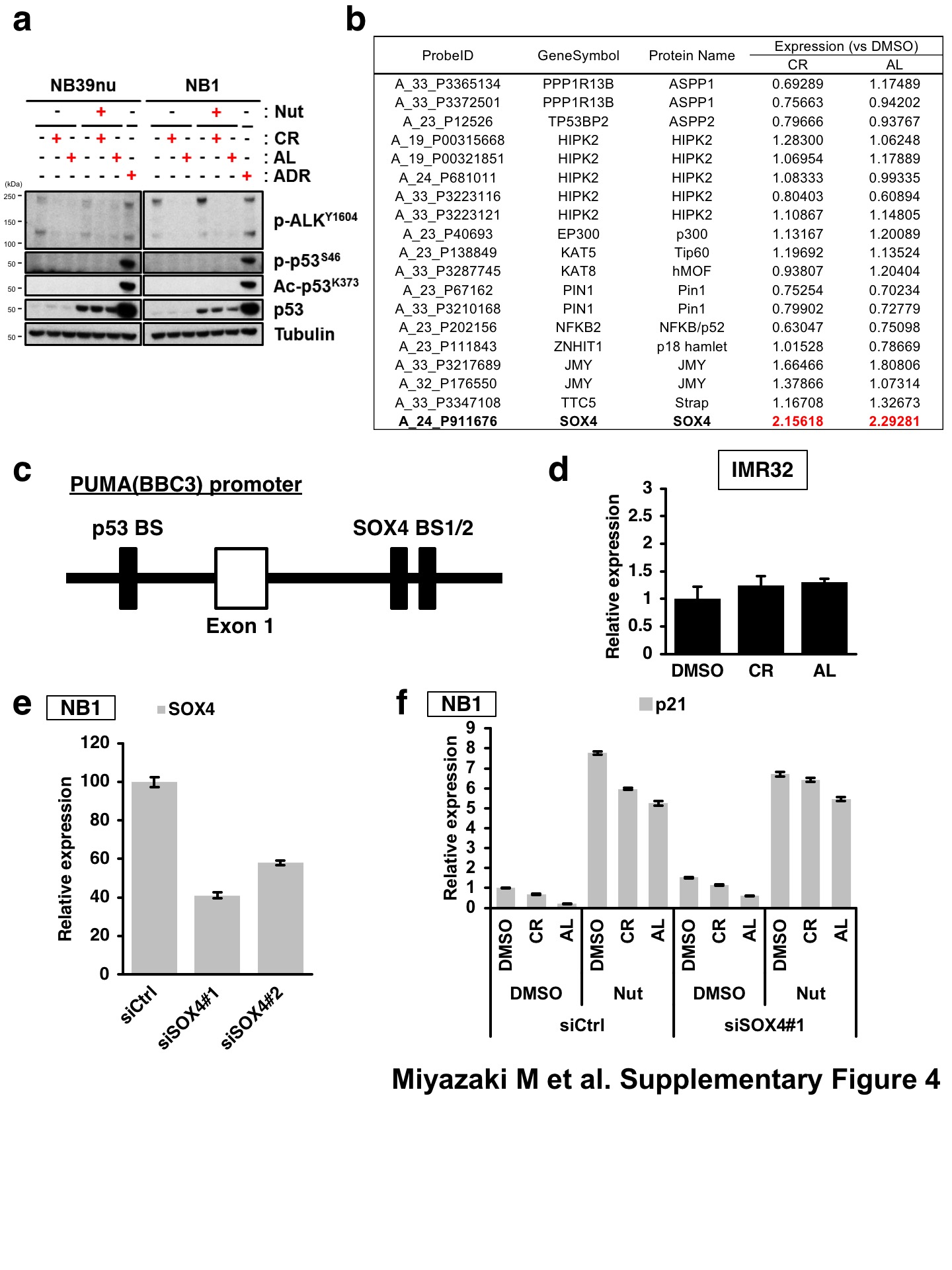


## **Supplementary Figure 4. The expression of SOX4 is up-regulated by ALK inhibitor. (Related to Figure 7)**

**a,** No effect of post-translational modification of p53 following combination treatment with ALK inhibitors and the p53 activator Nutlin-3. NB cells were treated with 1000 nM of either of the ALK inhibitors, 10 µM Nutlin-3, and 0.3 µM of adriamycin (ADR) for 16 h. An immunoblot analysis was carried out using the indicated antibodies. **b,** Both ALK inhibitors induce the up-regulation of SOX4. Changes in the levels of p53-binding partner that contribute to the p53-mediated transcriptional selectivity by the ALK inhibitors are shown. **c,** Structure of the PUMA promoter. The white box indicates exon 1, and the black boxes indicate the binding sites for p53 and SOX4. **d,** Expression of SOX4 in IMR32 cells. IMR32 cells were treated with the two ALK inhibitors (1 μM), as indicated, for 24 h. The expression of SOX4 was determined by qRT-PCR analysis. **e,** Knockdown efficiency of SOX4 by siRNA in MB39-nu and NB1 cells. The expression of SOX4 was determined by qRT-PCR analysis. **f,** NB1 cells were transfected with the siRNA targeting SOX4, and were treated with 1000 nM of either of the two ALK inhibitors, along with 10 µM Nutlin-3 for 16 h. The expression of p21 was then determined by qRT-PCR analysis. All data show the mean +/- SD (n = 3).

##
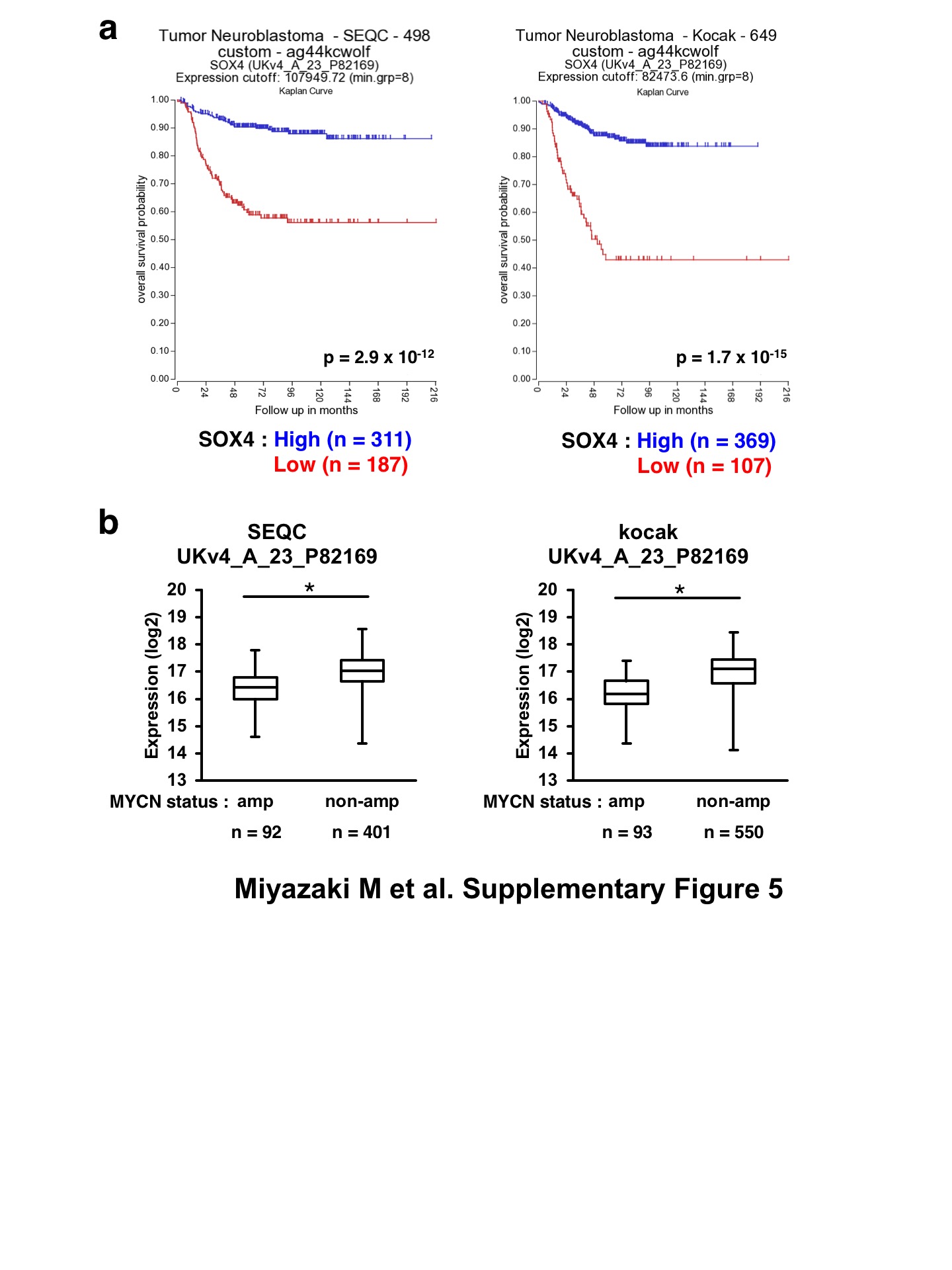


## **Supplementary Figure 5. The expression level of SOX4 is correlated with poor prognosis in neuroblastoma. (Related to Figure 7)**

**a,** Correlation of SOX4 expression and neuroblastoma prognosis was analysed using an R2 Genomics Analysis and Visualization Platform (http://r2.amc.nl). Two data sets (SEQC and Kocak) are used for the Kaplan-Meier plot. The p value was determined using a log-rank test. **b,** SOX4 expression from the two data sets in MYCN-amplified neuroblastomas compared with non-amplified neuroblastomas. *p < 0.05 (Welch’s t-test).

##
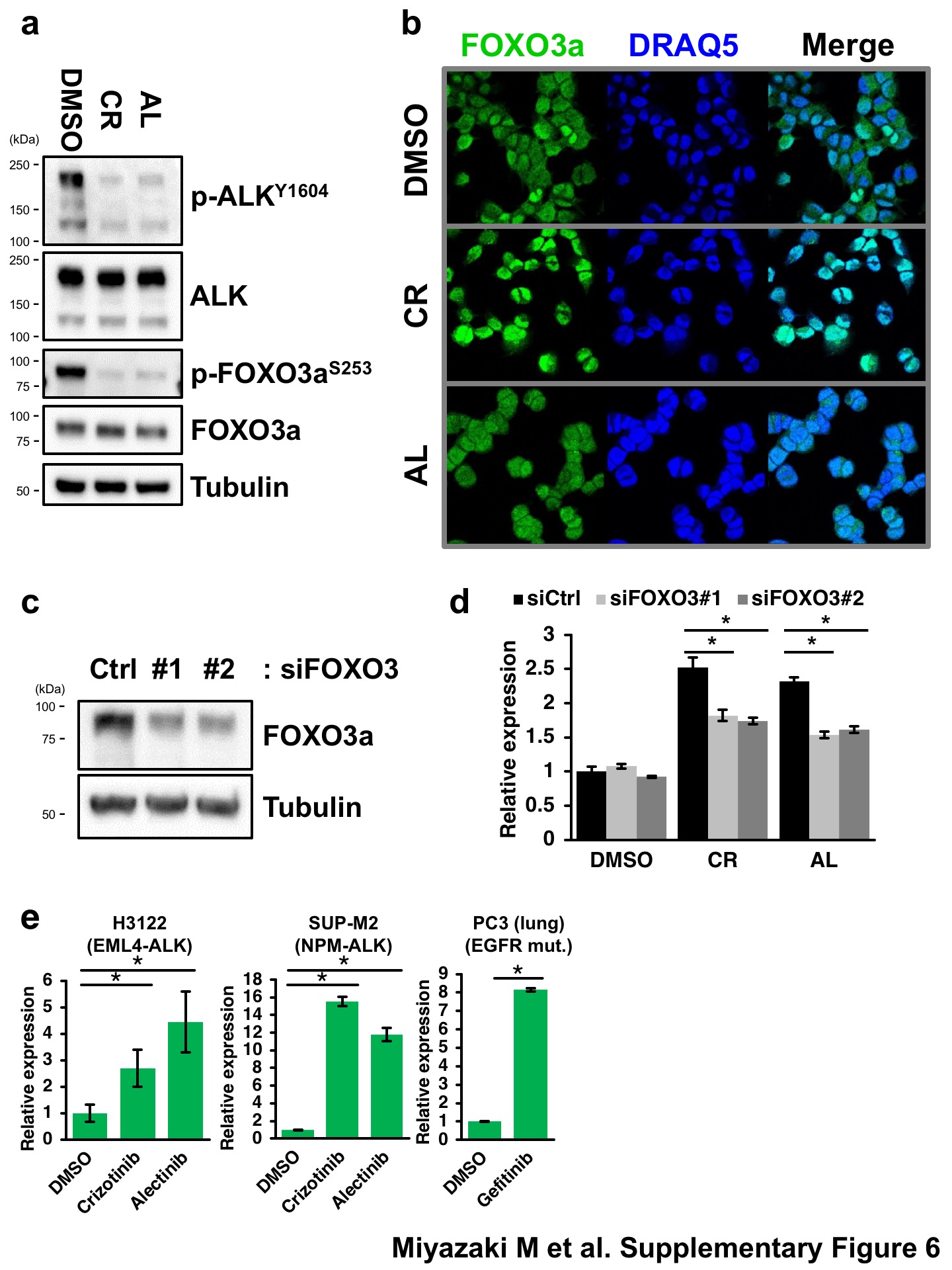


## **Supplementary Figure 6. ALK regulates SOX4 via the AKT-FOXO3a axis (Related to Figure 7).**

**a,** Dephosphorylation of FOXO3a by treatment with ALK inhibitors. NB1 cells were treated with 1000 nM of either of the two ALK inhibitors for 24 h, as indicated. An immunoblot analysis was conducted using the indicated antibodies, as shown. **b,** Nuclear translocation of FOXO3a elicited by the two ALK inhibitors. NB1 cells were treated with 1 μM of either of the two ALK inhibitors for 24 h, as indicated. DRAQ5 immunofluorescence was used for counter staining of the nuclei. **c, d,** Knockdown of FOXO3a abolishes the up-regulation of SOX4 by the ALK inhibitor. The knockdown efficiency of two FOXO3 siRNAs was confirmed by immunoblot analysis (c). After transfection with siRNA, NB1 cells were treated with 1000 nM of either of the ALK inhibitors for 24 h, as indicated. The expression of SOX4 was then determined by qRT-PCR analysis (d). **e,** Up-regulation of SOX4 by tyrosine kinase inhibitor. EML4-ALK positive lung cancer cells (H3122), NPM-ALK positive anaplastic large cell lymphoma cells (SUP-M2), and EGFR-mutated lung cancer cells (PC3) were treated with the indicated ALK inhibitors and the EGFR inhibitor, gefitinib (all 1 μM). The expression of SOX4 was determined by qRT-PCR analysis. The data are shown as mean +/- SD (n = 3). *p < 0.05.

##
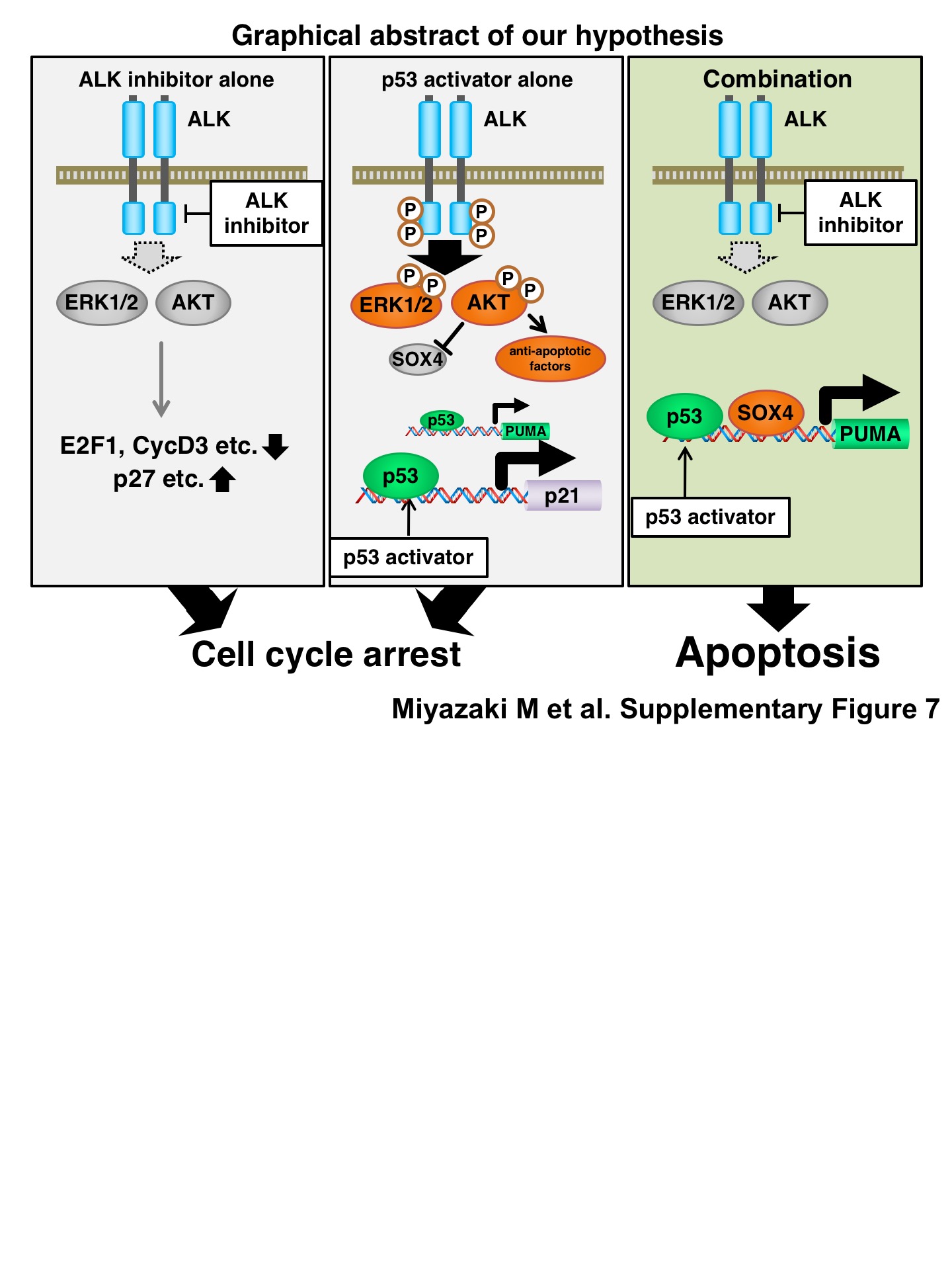


## **Supplementary Figure 7. The mechanism underlying irreversible cell growth following combination treatment of an ALK inhibitor with a p53 activator.**

In the case of the ALK inhibitor alone, the ALK inhibitor suppresses ALK and its downstream signalling pathways, which leads to cell-cycle arrest accompanied by the down-regulation of cell-cycle-promoting factors, including E2F1 and CycD3, and the up-regulation of cell-cycle inhibitory factors including p27. In the case of the p53 activator alone, the p53 activator stabilizes p53 levels and predominantly induces p53-mediated cell-cycle-arrest through increased p21 expression, rather than the induction of p53-mediated pro-apoptotic PUMA. At this time, anti-apoptotic factors are presumably up-regulated by ALK and consequently, cells resistant to apoptosis survive. However, in the case of the combination treatment, the ALK-mediated anti-apoptotic pathways are inactivated and SOX4, increased as a result of the inactivation of the ALK-AKT-FOXO3a signalling pathway, enhances p53-mediated PUMA expression, eventually leading to increased apoptosis.
